# Macroalgal microbiomes unveil a valuable genetic resource for halogen metabolism

**DOI:** 10.1186/s40168-023-01740-6

**Published:** 2024-03-07

**Authors:** Anna Lavecchia, Bruno Fosso, Aschwin H. Engelen, Sara Borin, Caterina Manzari, Ernesto Picardi, Graziano Pesole, Antonio Placido

**Affiliations:** 1 Department of Biosciences, Biotechnology and Environment, University of Bari “Aldo Moro”, Via Orabona 4, Bari, 70124 Italy; 2 Center of Marine Sciences (CCMar), University of Algarve, Campus Gambelas, Faro, 8005-139 Portugal; 3 Department of Food, Environmental and Nutritional Sciences, University of Milan, Via Celoria 2, Milan, 20133 Italy; 4 Institute of Biomembranes, Bioenergetics and Molecular Biotechnologies, National Research Council of Italy, Via Giovanni Amendola, Bari, 122/O, 70126 Italy

**Keywords:** Holobiont, Macroalgae, *Sphaerococcus coronopifolius*, *Asparagopsis taxiformis*, *Halopteris scoparia*, Microbiome, Metagenome-assembled genomes (MAGs), Organohalogens, Halogenation, Dehalogenation

## Abstract

**Background:**

Macroalgae, especially reds (Rhodophyta Division) and browns (Phaeophyta Division), are known for producing various halogenated compounds. Yet, the reasons underlying their production and the fate of these metabolites remain largely unknown. Some theories suggest their potential antimicrobial activity and involvement in interactions between macroalgae and prokaryotes. However, detailed investigations are currently missing on how the genetic information of prokaryotic communities associated with macroalgae may influence the fate of organohalogenated molecules.

**Results:**

To address this challenge, we created a specialized dataset containing 161 enzymes, each with a complete enzyme commission number, known to be involved in halogen metabolism. This dataset served as a reference to annotate the corresponding genes encoded in both the metagenomic contigs and 98 metagenome-assembled genomes (MAGs) obtained from the microbiome of 2 red (*Sphaerococcus coronopifolius* and *Asparagopsis taxiformis*) and 1 brown (*Halopteris scoparia*) macroalgae. We detected many dehalogenation-related genes, particularly those with hydrolytic functions, suggesting their potential involvement in the degradation of a wide spectrum of halocarbons and haloaromatic molecules, including anthropogenic compounds. We uncovered an array of degradative gene functions within MAGs, spanning various bacterial orders such as *Rhodobacterales*, *Rhizobiales*, *Caulobacterales*, *Geminicoccales*, *Sphingomonadales*, *Granulosicoccales*, *Microtrichales*, and *Pseudomonadales*. Less abundant than degradative functions, we also uncovered genes associated with the biosynthesis of halogenated antimicrobial compounds and metabolites.

**Conclusion:**

The functional data provided here contribute to understanding the still largely unexplored role of unknown prokaryotes. These findings support the hypothesis that macroalgae function as holobionts, where the metabolism of halogenated compounds might play a role in symbiogenesis and act as a possible defense mechanism against environmental chemical stressors. Furthermore, bacterial groups, previously never connected with organohalogen metabolism, e.g., *Caulobacterales*, *Geminicoccales*, *Granulosicoccales*, and *Microtrichales*, functionally characterized through MAGs reconstruction, revealed a biotechnologically relevant gene content, useful in synthetic biology, and bioprospecting applications.

Video Abstract

**Supplementary Information:**

The online version contains supplementary material available at 10.1186/s40168-023-01740-6.

## Background

Marine microorganisms are still largely unexplored in many aspects, yet, they represent an extraordinary source of genes, enzymes, and metabolites. These resources have significant potential to encourage the development of novel biotechnological and medical applications, while also being valuable for investigating marine ecosystems and ecological processes [1–6].

Currently, the exploration of metagenome-assembled genomes (MAGs) represents a powerful approach to identify new candidate taxa from uncultivable prokaryotes and clarify their ecological features and potential applications [7–10]. The Tara Oceans Expeditions produced over 2500 bacterial and archaeal MAGs derived from more than 200 marine metagenomic samples. Phylogenomic analysis of these MAGs revealed lineages that had no previously cultured representatives [11]. Nearly 8000 high-quality MAGs of uncultivated bacteria and archaea (UBA) were recovered from over 1500 publicly available metagenomes, noticeably expanding the Earth’s prokaryotic genomic diversity [12].

Among marine multicellular hosts, macroalgae function as a foundation species playing a key role in shaping microbial communities by providing a physical structure and engaging in mutualistic exchanges [13]. Like plants, macroalgae can also be considered holobionts, consisting of the multicellular host and its associated microbial community. In this symbiotic relationship, each partner actively contributes to the maintenance, performance, and resilience of the holobiont [14]. The first studies including prokaryote-macroalga interactions based on MAGs analysis have been published only recently. A comparative genome-centric analysis of reconstructed MAGs from *Sargassum* spp. macroalgae biofilms revealed that variations in the function of coral reef microbiomes were influenced by seasonality, macroalga biomass abundance, and available nutrients [15]. Another recent study explored the relationship between the kelp *Nereocystis luetkeana* and its microbiome. Genes involved in moving and assimilating abundant dissolved organic matter produced by kelp were annotated in various microorganisms composing the *Nereocystis luetkeana* microbiome. This discovery strengthens the concept of the holobiont [16].

Macroalgal–prokaryotic interactions are dependent on the production of different chemical compounds [13]. Notably, halogenated compounds produced by both macroalgae and associated prokaryotes play a significant role in regulating these mutual interactions [17]. Red and brown marine macroalgae are well known to synthesize halogen-containing compounds, which are believed to play a role in regulating biofouling on the algae’s surface, as well as grazing and defending against pathogens [18–22]. Nonetheless, little is known about the metabolic interconnection between the host and microbiota concerning the production and degradation of these compounds. Prokaryotes associated with macroalgae must tolerate the antimicrobial effects of organohalogens synthesized by their host. They achieve this by possessing genes that encode for enzymes capable of managing various halogenated compounds. Conversely, macroalgae-associated microorganisms equipped with biosynthetic functions could enhance their own competitiveness and provide an advantage to the host, shaping the microbial community and controlling the proliferation of harmful taxa. In this contest, MAGs from red and brown marine macroalgae could serve as a valuable genetic resource. They provide new means to explore the complex network of prokaryotic taxa and enzymes involved in biosynthetic and degradative halogenation processes, ultimately aiding in unraveling marine ecological interactions.

Naturally occurring halogenated compounds possess significant medical and industrial value, displaying a wide range of biological activities [23–25]. For instance, the process of biofluorination carried out in engineered *Pseudomonas putida* is a clear example of synthetic biology [26]. This approach aims to achieve economically feasible and environmentally sustainable production of organohalides [27]. The use of GenoChemetic methods, using genes encoding heterologous halogenases, is a developing approach to generate analogs of natural halogenated products. These derivatives play a crucial role in medicine and agriculture [25]. Genes encoding enzymes involved in dehalogenation pathways are therefore pivotal for developing or improving important biotechnological applications [28–30].

Considering the production of antimicrobial halogenated compounds by macroalgae, our proposal is to investigate the hypothesis that a large proportion of their closely associated bacteria may possess a range of protein-coding genes capable of metabolizing organohalogens. This potential ability might serve as a driving factor in shaping the structure of seaweed holobionts. At the same time, we aim to reveal seaweed microbiomes as a valuable future biotech source. Therefore, we deeply sequenced the epiphytic metagenomes of 2 red (Rhodophyta Division) and 1 brown (Phaeophyta Division) marine macroalgae: *Sphaerococcus coronopifolius* (*Sc*), *Asparagopsis taxiformis* (*At*), and *Halopteris scoparia* (*Hs*). This data enabled the assembly of a total of 98 prokaryotic draft genomes, uncovering novel taxa within each macroalga. Moreover, we detected several genes encoding for enzymes related to halo- and dehalogenation metabolism, annotated using a specialized reference dataset of functions. This exploration revealed both biosynthetic capabilities and a significant biotechnological potential for hydrolytic dehalogenation. To the best of our knowledge, this is the first report describing a genetic resource potentially involved in organohalogen metabolism within microbiomes associated with multicellular eukaryotic organisms generating organohalogens.

## Methods

### Macroalgae sampling, epiphytic metagenomic DNA extraction, shotgun sequencing, and MAGs detection

Three composite samples of algae (one from each macroalga, consisting of at least three distinct individuals) were collected from the same location, at the harbor bay of Lagosteiros (37° 1′ 9.678′′ N 7° 55′ 49.584′′ W), in southwest Portugal. These macroalgae were sampled from the shallow intertidal area via snorkeling in October 2016. The samples were kept refrigerated for 24 h and transported on ice to the laboratory. Subsequently, they were rinsed 3 times in a sterile Petri dish using artificial seawater filtered through a 0.2 µm filter to remove loosely attached microbes. Using a 50-ml sterile tube, a short centrifugation (500 × g, 2 min per 2 times; Eppendorf 5417R, Hamburg, Germany) was done to remove the remaining seawater from tissue samples. These samples were subsequently stored at −80 °C until DNA extraction.

Metagenomic DNA was extracted from the thawed algal tissue using the kit Quick-DNA™ Fecal/Soil Microbe Midiprep (Zymo Research, Irvine, CA, USA). We followed the recommended guidelines for sample processing (2.5 g per macroalgal composite sample in the present study, a mix including ca. 0.85 g per single individual). However, instead of using a bead beater, we opted to vigorously mix the samples with a vortex (Scientific Industries, Bohemia, NY, USA.) for 10 min at maximum speed. This method was intended to minimize the breakdown of algal tissue and reduce the risk of potential contamination from host genomic DNA. Quantitative PCRs were then performed using serial decimal dilutions of extracted DNA as a template and a specific couple of primers targeting regions of the bacterial 16S rRNA (following the protocol by Mapelli et al. [31]) and seaweed 18S rRNA (following the protocol by Herrero et al. [32]) genes.

One metagenome for each algal species was analyzed for a qualitative comparison among the three microbiomes. No technical and biological replicates were generated.

The DNA libraries for all three metagenomic DNA were prepared following the TruSeq Nano DNA library prep workflow (Illumina, Illumina San Diego, CA, USA), adhering to the manufacturer’s instructions. Subsequently, the three libraries were sequenced on an Illumina NextSeq 500 platform, generating 2 × 75 bp paired-end reads.

Raw reads were quality-trimmed, and the adapters were removed by using trim-galore! v0.6.4 (https://www.bioinformatics.babraham.ac.uk/projects/trim_galore/). The retained reads were assembled in contigs by using metaSPAdes v3.13.1 [33] and binned in MAGs by MetaBAT2 v2.13 [34]. CheckM v1.0.18 [35] was finally applied to evaluate MAGs completeness and contamination. The raw sequencing data and associated metadata have been deposited and can be accessed under the NCBI BioProject PRJNA915238.

### Taxonomy and phylogeny

The CheckM ssu_finder script was used to detect prokaryotic SSU rRNA genes within the obtained contigs, and SSU ≥ 300nt were taxonomically annotated in BioMaS [36]. This annotation was done using the release 138 NR 99 of the SILVA databases [37]. Chord diagrams of the taxa shared among the macroalga microbiomes were obtained by using the circlize R library [38].

For *Asparagopsis taxiformis*, an evaluation of the host genome contamination was performed by mapping trimmed reads on the host genome originating from Guam (JAAEFF000000000.1) through bowtie2 [39]. For the other two macroalgae, no reference genome is available.

The taxonomic classification of all high- and medium-quality MAGs was performed by using KBase implemented GTDB-Tk version 1.7.0 (GTDB R06-RS202 release (https://narrative.kbase.us/#catalog/apps/kb_gtdbtk/run_kb_gtdbtk_classify_wf/release) [9, 40] a software toolkit for assigning objective taxonomic classifications to bacterial and archaeal genomes based on the GTDB [41, 42] constructed from RefSeq and Genbank genomes.

Phylogenetic relatedness among all MAGs was established by SpeciesTreeBuilder v.0.1.3 (https://narrative.kbase.us/#catalog/modules/SpeciesTreeBuilder) in KBase. This process relied on FastTree2 (version 2.1.10) [43] and employed a set of 49 core, universal genes defined by Clusters of Orthologous Groups (COG) gene families. The resulting phylogenetic trees, in Newick format, were then imported into the interactive Tree of Life (iTOL) version 6.6 [44] for visualizing the phylogenetic relationships among the assembled genomes and their associated functional profiles.

### Developing BRENDA-EC161 and -EC42 dataset

The enzyme commission serial numbers (EC X.X.X.X) for all enzymes involved in halogen metabolism were retrieved from BRENDA (Braunschweig Enzyme Database, https://www.brenda-enzymes.org) using both the explorer (https://www.brenda-enzymes.org/ecexplorer.php) and full-text search (https://www.brenda-enzymes.org/fulltext.php) functions. The search terms included “halo”, “halides”, “dehalogenase”, and “halogenase”. When necessary, specific source links from KEGG (Kyoto Encyclopedia of Genes and Genome), MetaCyc (Metabolic Pathways From all Domains of Life), and UniProtKB (UniProt Knowledgebase) were used for additional queries. The enzymes that are specifically involved in halo- or dehalogenation reactions and are fully categorized by an EC number are reported in Additional file 2: Table S1.

There are many degradative and biosynthetic reactions involving organohalogens, whose enzymatic components have not been fully characterized, resulting in an incomplete assignment of EC numbers (e.g., brominated pyrroles biosynthesis pathway, https://metacyc.org/META/NEW-IMAGE?type=PATHWAY&object=PWY-7931). Therefore, enzymes with partially assigned EC numbers were not considered because they were not fully characterized.

### Metagenomes and MAGs functional annotation

The metagenomic contigs and MAGs were annotated using Prokka v1.14.5 [45], a tool which is available in the DOE Systems Biology Knowledgebase (KBase) platform. KBase is an open-source software and data platform designed for predicting and designing biological functions within microbial communities (https://www.kbase.us/). Default parameters were used in the Prokka annotation process. The resulting feature tables (.tsv format) were then used to extract all annotated EC numbers, from which 161 EC numbers were selected.

For both, single BRENDA target function and clusters, the gene density was estimated as a ratio between the number of annotated genes and the total size (Mbp) of assembled metagenomic contigs.

## Results

### Macroalga microbiomes

The three macroalga species (*Sc*, *At*, and *Hs*) were sampled from the same Portugal continental coastal area. The primary goal was to minimize contamination originating from the host genome, thus aiming to obtain a metagenome that predominantly contains DNA from epiphytic microbiota. By the quantitative PCRs performed, no algal 18S rRNA amplicons were detected on all the three extracted metagenomic DNA samples up to 10,000-fold DNA dilutions, while 16S rRNA amplicons were observed (data not shown). Consistently, the host *At* 18S rRNA gene was detected by whole shotgun metagenomic sequencing and annotated (Additional file 1: Table S1) but the DNA genomic contamination of such host was very low (2.93%) (see the “Methods” section), confirming the validity of the extraction method. Unfortunately, the estimation of *Sc* and *Hs* genome contamination were not performed due to the unavailability of reference genomes within public genome databases. The extraction of some endophytic microbial genomes is not excluded considering the weak host DNA contamination.

The total size of metagenomic assembled contigs obtained after the assembly of reads were 784.246.579 bp for *Sc*, 525.823.578 bp for *At*, and 435.330.334 bp for *Hs*. For detailed sequencing metadata (e.g., number of reads and contigs), see Additional file 1: Table S2.

### Phylogenetic diversity of microbiomes

**Fig. 1:**
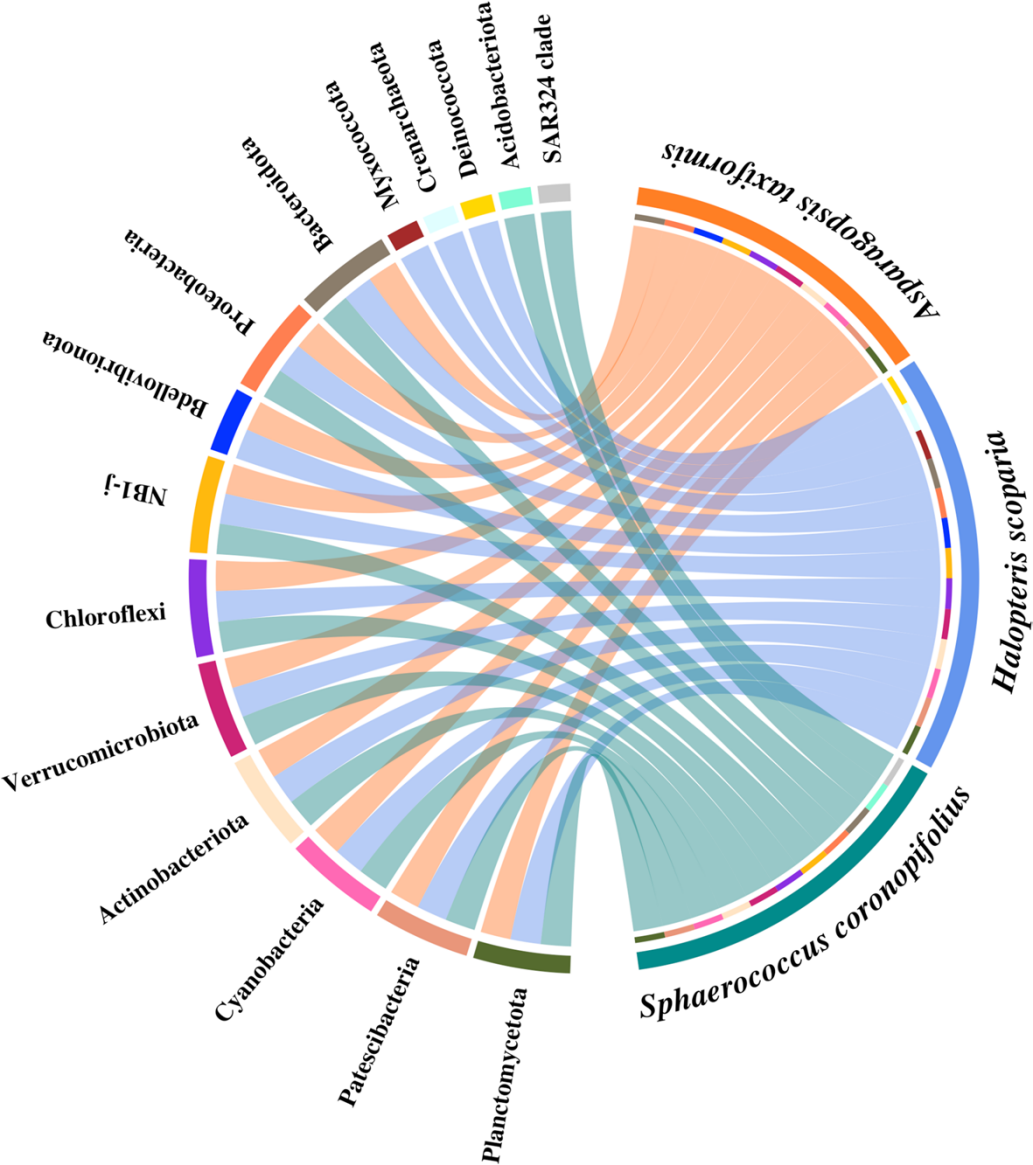
Chord diagram showing the observed prokaryotic phyla based on the taxonomic classification of the SSU rRNA sequences identified in the metagenomic contigs. It illustrates their distribution among the macroalgae-associated microbiomes. Inside the circle: in green the phyla assigned to *Sphaerococcus coronopifolius*, in blue and in orange those assigned to *Halopteris scoparia* and *Asparagopsis taxiformis*, respectively

An initial taxonomic overview of phylogenetic diversity of the microbiomes associated with the three investigated macroalgae, using all contigs encoding SSU rRNA sequences showed a phylogenetic diversity consisting of 10, 11, and 13 phyla in *At*, *Sc*, and *Hs* contigs, respectively (Fig. 1 and Additional file 1: Table S1). The three algae shared 9 phyla.

The SSU rRNAs assigned to *Proteobacteria* and *Bacteroidota* phyla were prevalent in all 3 hosts. Exclusive phyla were found in *Sc* (*Acidobacteriota* and SAR324 group B) and *Hs* (*Crenarchaeota*, *Deinococcota*, and *Myxococcota*), while they were absent in *At*. The phylum *Bdellovibrionota* was represented in both *At* and *Hs* contigs. Only one phylum belonging to Archaea, specifically *Hs Crenarchaeota*, was detected.

At the order level, the SSU rRNA gene analysis revealed a more evident taxonomic diversity across all 3 microbiomes. In *Hs*, *Sc*, and *At*, contigs of 32, 29, and 28 orders were observed, respectively (Additional file 2: Fig. S1). *Hs* revealed more exclusive orders (10), followed by *Sc* (6) and *At* (6). A further remarkable bacterial diversity was observed at the genus level, as well as a remarkable specificity of taxa represented in the three algae (Additional file 2: Fig. S2).

### Taxonomy of MAGs

Of the over 200 MAGs reconstructed (Additional file 1: Table S3), we retained a total of 98 high- (completeness > 90% and contamination < 5%) and medium-quality MAGs (completeness ≥ 50% and contamination < 10%): 49 from *Sc* (7 high- and 42 medium-quality), 31 from *At* (10 high- and 21 medium-quality), and 18 from *Hs* (3 high- and 15 medium-quality) (Additional file 1: Table S4).

The obtained MAGs, classified by using GTDB-Tk (Additional file 1: Table S5), exhibited globally a phylogenetic diversity of 11 phyla (Additional file 2: Fig. S3). Among the 3 MAGs sets, *Sc*, *Hs*, and *At* displayed 10, 6, and 3 phyla, respectively. One medium-quality genome (MAG32) from *Hs* could not be assigned to any prokaryotic domain (bacteria or archaea). Overall, the phyla assigned to the MAGs were generally consistent with those found in the contigs. However, discrepancies were found, such as the presence of *Bdellovibrionota* and *Myxococcota* only in *Sc* MAGs and absent in the contigs. Conversely, NB1-j, SAR324 clade-Marine group B, and *Planctomyceta* were exclusively detected in the contigs and not in the MAGs. At the class level, *Bacteroidia*, *Alphaproteobacteria*, and *Gammaproteobacteria* were the most represented, with 27 MAGs (11 *Sc*, 9 *At*, 7 *Hs*), 23 MAGs (11 *Sc*, 12 *At*, 0 *Hs*), and 21 MAGs (8 *Sc*, 8 *At*, 5 *Hs*), respectively (Additional file 2: Fig. S4). However, *Alphaproteobacteria* MAGs were not detected in the assembly of *Hs* contigs. At the order level, *Flavobacteriales* were the most represented with 24 MAGs (10 *Sc*, 8 *At*, 6 *Hs*), followed by *Sphingomonadales* (3 *Sc*, 3 *At*, 0 *Hs*), *Pseudomonadales* (3 *Sc*, 1 *At*, 2 *Hs*), *Enterobacterales* (1 *Sc*, 4 *At*, 1 *Hs*), and *Caulobacterales* (2 *Sc*, 4 *At*, 0 *Hs*) each accounting for 6 MAGs each (Additional file 2: Fig. S5). The remaining MAGs were distributed among 23 additional orders each containing between one to five MAGs. *Flavobacteriales*, *Pseudomonadales*, and *Enterobacterales* MAGs were observed across all macroalgae.

Out of the 98 MAGs under examination, GTDB-Tk taxonomic placement criteria (considering topology, relative evolutionary divergence, and average nucleotide identity) assigned 90 MAGs into novel ranks: 6 as novel families, 31 as novel genera, and 53 as novel species (Additional file 1: Tables S5 and S6). Seven MAGs were assigned to known species, whereas *Hs*MAG32 could not be classified. Using a phylogenetic tree based on Clusters of Orthologous Groups of proteins (COG) constructed with FastTree2 (Additional file 2: Fig. S6) showed that *Hs*MAG32 represents an independent prokaryotic clade belonging to the Archaea domain related to the candidate genus *Nitrosopumilus*. Further analysis of the SSU rRNA gene analysis within the *Hs* metagenome revealed the presence of contigs belonging to the phylum *Crenarchaeota* (*Candidatus_Nitrosopumilus* genus) (Additional file 1: Table S1).

### Dataset of BRENDA enzymes active in the organohalogen metabolism

To annotate the genetic pool encoding enzymes involved in organohalogen metabolism within *At*, *Sc*, and *Hs* metagenomes and MAGs, a custom dataset composed of 161 BRENDA enzymes, each with a complete EC number, was collected (Additional file 2: Table S1). This dataset served as a reference for the annotation process. For each enzyme, details such as the recommended name, the IUBMB, KEGG, MetaCyc, and UniProtKB annotations related to the catalyzed reactions and target metabolic pathways were provided.

Out of the 161 enzymes (hereinafter called BRENDA-EC161 functions), 42 are directly involved in the genesis or breaking of halide bonds: 16 halogenases and 26 dehalogenases. Hereafter, these 42 enzymes will be referred to as BRENDA-EC42 functions. The remaining 119 enzymes are not directly involved in the genesis or breaking of halogen bonds but play a role in the chemical transformations that precede or follow the halogenation or dehalogenation of substrates.

### Biodegradative and biosynthetic potential of BRENDA target genes in the analyzed metagenomes

**Fig. 2:**
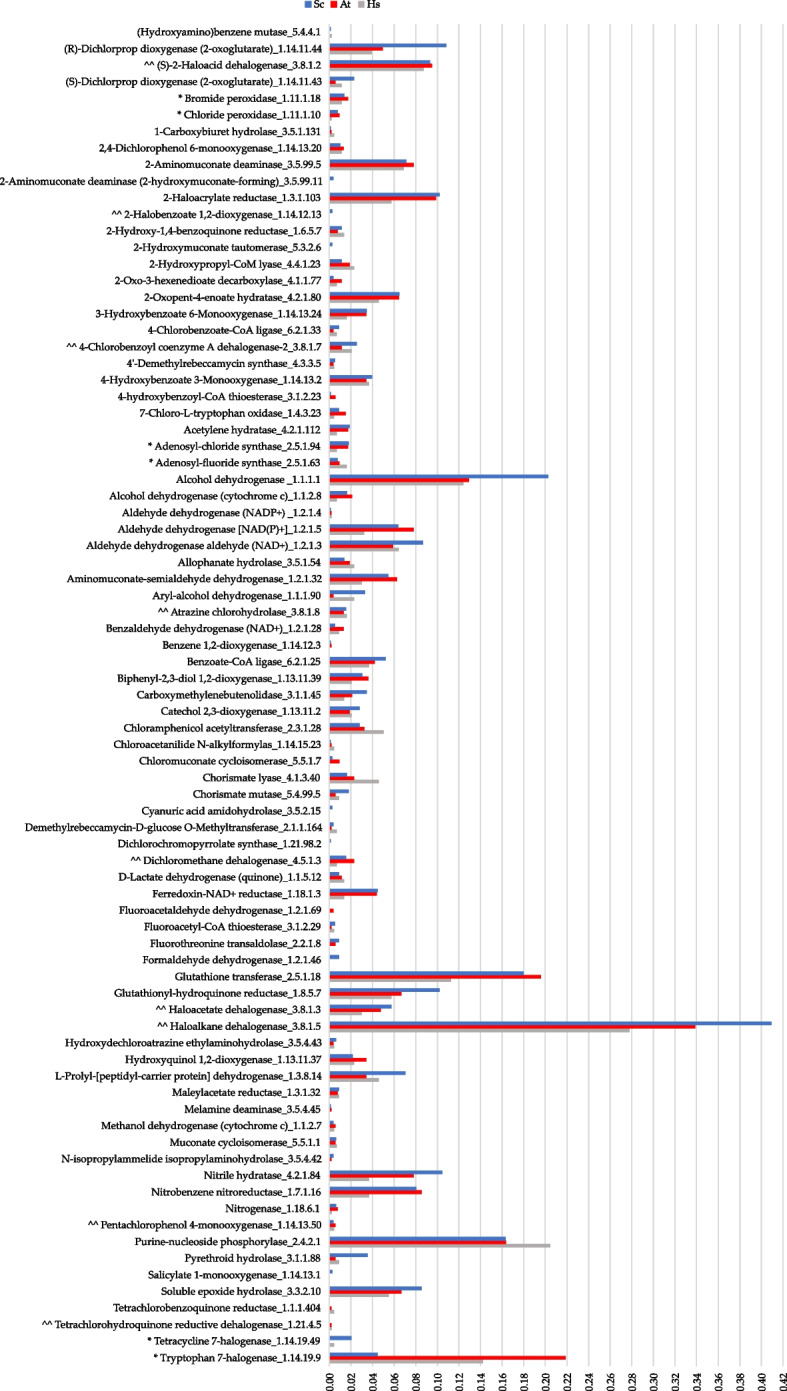
Gene density values of target genes annotated within the *Sphaerococcus coronopifolius* (*Sc*, blue bar), *Asparagopsis taxiformis* (*At*, red bar), and *Halopteris scoparia* (*Hs*, gray bar) sequenced metagenomes. The list indicates the recommended name of the 81 encoded BRENDA enzymes and their associated complete EC number (X.X.X.X). The list shows the enzymes considering enantiomers (*R*/*S*), substituent numerical position, and alphabetical order. Annotated BRENDA-EC42 subgroup functions: ^^ dehalogenases, * halogenases, and gene density values (genes/Mbp)

Using the BRENDRA-EC161 reference collection, a total of 81 functions were annotated across the three metagenomes, with 17 and 64 related to halogenation and dehalogenation pathways, respectively (Additional file 2: Table S2). Most of the functions were shared among the metagenomes (63 out of 81, 77.8%), and only 18 were retrieved in one (*Sc* or *At*) or two metagenomes (*Sc*-*At*, *Sc*-*Hs*, or *At*-*Hs*). Genes encoding haloalkane dehalogenases (EC 3.8.1.5) were the most abundant in all metagenomes. The gene density value (expressed as genes/Mb) for this hydrolytic enzyme was 0.41 in *Sc*, 0.34 in *At*, and 0.27 in *Hs* (Fig. 2 and Additional file 1: Table S7A).

A total of 15 BRENDA-EC42 subgroup functions were annotated of which 12 (80%) were shared among the 3 metagenomes (Fig. 2 and Additional file 2: Table S2). Genes encoding 5 different hydrolytic dehalogenases (EC 3.8.1.X) formed the most conspicuous functional group directly involved in the breaking of carbon-halogen bonds. The tryptophan 7-halogenase (EC 1.14.19.9) gene was the most recurrent in halide bond genesis and was observed in all three metagenomes. The gene density value was particularly high in *At* (0.22) and *Hs* (0.14) compared with *Sc* (0.04) (Fig. 2 and Additional file 1: Table S7).

**Fig. 3:**
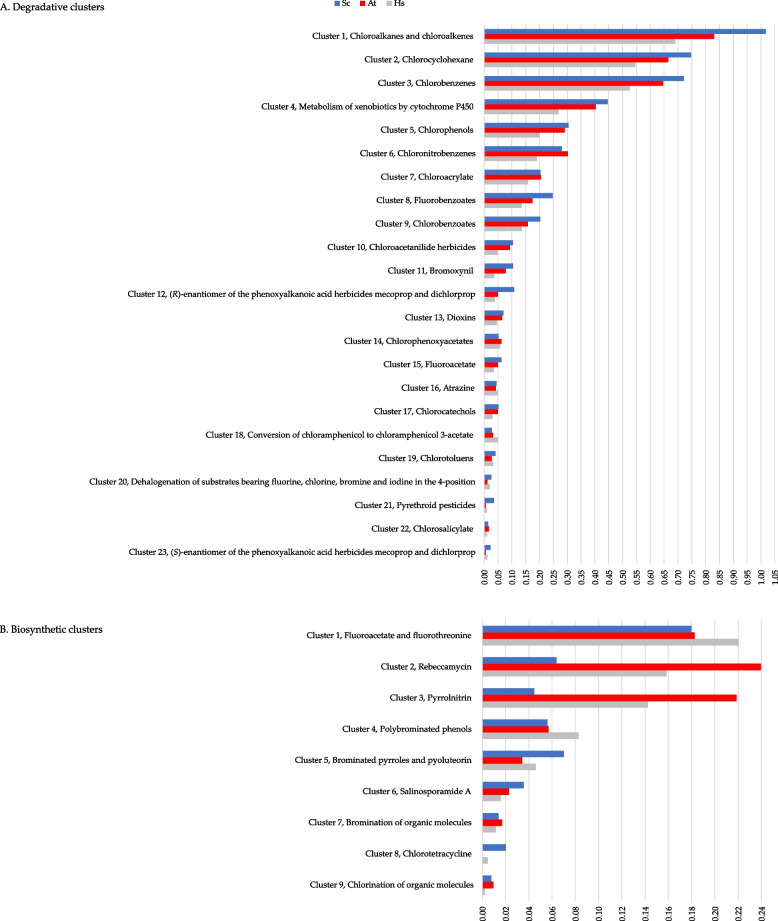
Gene density values for potential degradative and biosynthetic clusters involved in halogen metabolism depicted for the *Sphaerococcus coronopifolius* (*Sc*, blue bar), *Asparagopsis taxiformis* (*At*, red bar), and *Halopteris scoparia* (*Hs*, gray bar) metagenomes. **A** Degradative gene clusters (numbered from 1 to 23) along with the chemical types of halogenated molecules degraded. **B** Biosynthetic gene clusters (numbered from 1 to 9) and chemical types of halogenated metabolites produced. The cluster gene density values are shown as genes/Mbp. The clusters are arranged in descending order of their density values

Based on IUBMB, KEGG, MetaCyc, and UniProtKB annotations (Additional file 2: Table S1), we clustered the 81 BRENDA annotated genes for each macroalga metagenome based on shared categories of degraded molecules or produced metabolites. Subsequently, we calculated the gene density per cluster (expressed as genes/Mbp) (Fig. 3 and Additional file 1: Table S7B). A total of 32 gene clusters were identified in the metagenomes, 23 involved in biodegradation (Fig. 3A), and 9 in biosynthetic processes (Fig. 3B). Thirty-one of the 32 clusters were found to be shared by all 3 metagenomes, indicating that three algal microbiomes shared a similar set of bacterial BRENDA-EC161 functions. The chlortetracycline biosynthetic gene set was not detected in *At*. Concerning the potential for organohalogens biodegradation, the highest gene densities were observed within the cluster including degradation reactions of chloroalkanes and chloroalkenes (*Sc*, 1.02; *At*, 0.83; *Hs*, 0.69) (cluster 1, Fig. 3A).

Cluster 1 revealed a remarkable hydrolytic potential for dehalogenation across all three macroalga metagenomes, especially in genes encoding haloalkane, haloacetate, and (*S*)-2-haloacid dehalogenases (Additional file 1: Table S7C). Furthermore, a remarkable gene density was also found within the cluster of genes responsible for the activity on chlorocyclohexane, a cyclic aliphatic haloalkane (Fig. 3A).

In addition, several benzene halocompounds appear to be among the main potential targets for degradation. Clusters including genes encoding enzymes responsible for breaking down different haloaromatic compounds, each containing a single benzene ring, were detected. Among them, a high gene density was recorded for the cluster (3) acting on chlorobenzenes. Following this, clusters 5, 6, 8, and 9 were observed to act on chlorophenols, chloronitrobenzenes, fluorobenzoates, and chlorobenzoates, respectively. This trend was notably evident in the two red algae, particularly in *Sc*. Genes involved in degrading benzene derivates were found to be shared among all 3 metagenomes, including chlorochatecols (cluster 17) and chlorotoluenes (cluster 19).

Gene clusters potentially active in the degradation of other types of halomolecules were also annotated. In particular, genes involved in chloroacrylate degradation (cluster 7; Fig. 3A). Within this cluster, the most represented genes encode for the 2-haloacrylate reductase and the (*S*)-2-haloacid dehalogenase, which are enzymes responsible for metabolizing chloroacrylate. Gene clusters involved in the degradation of xenobiotics (cluster 4), herbicides (clusters 10, 11, 12, 14, 16, and 23), dioxins (cluster 13), antimicrobials (clusters 15 and 18), pyrethroid pesticides (cluster 21), and chlorosalicylate (cluster 22) were detected in all three metagenomes. Among these, cluster 4, comprising genes encoding for cytochrome P450 family enzymes and glutathione transferases, showed the highest density (Additional file 1: Table S7C).

Considering the 9 biosynthetic clusters, gene density values tended to be lower compared to the degradative ones (Fig. 3). A notable density value was observed for the *At* cluster involved in biosynthetic reactions for producing the antibiotic rebeccamycin (0.24) (cluster 2). Instead, the cluster associated with *Hs* chlorination reactions of organic molecules showed a lower density value (0.002) (cluster 9). High-density values were also observed for the *Hs* and *At* clusters involved, respectively, in the synthesis of fluoroacetate and fluorothreonine (cluster 1) as well as pyrrolnitrin (cluster 3), both antibiotics.

Other clusters involved in additional antibiotic biosynthesis were also found: pyoluteorin (cluster 5), salinosporamide A (cluster 6), and chlortetracycline (cluster 8). Cluster 5 also included the biosynthesis of brominated pyrroles. Concerning the biosynthesis of other organohalogens, cluster 4 resulted to be potentially active in the genesis of polybrominated phenols, whereas clusters 7 and 9 were associated with the bromination and chlorination of organic molecules, respectively.

### Phylogeny of MAGs and taxonomic distribution of BRENDA target functions along the cladogram

**Fig. 4:**
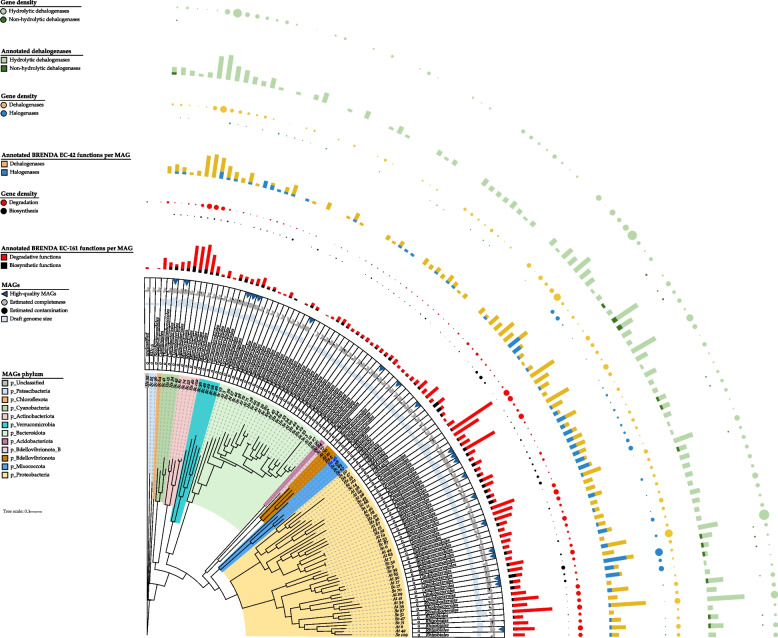
Phylogenetic tree showing the relationships between the 98 MAGs and the distribution of BRENDA-EC161 functions density annotated for each MAG, particularly dehalogenases and halogenases (BRENDA-EC42) and hydrolytic and non-hydrolytic dehalogenases gene densities. The 2 phyla including clades enriched in BRENDA-EC161 functions are shown in pink (*Actinobacteriota*) and light orange (*Proteobacteria*). MAGs assigned orders are also indicated. (detailed gene density values are in Additional file 1: Table S8). Ref* indicates an unsupervised reference genome of the public KBase genomes database used in phylogenetic tree building. *Sc*, *Sphaerococcus coronopifolius*; *At*, *Asparagopsis taxiformis*; *Hs*, *Halopteris scoparia*

The phylogenetic relationships among the 98 MAGs as well as the taxonomic distribution of their annotated BRENDA target functions and related gene density are shown in Fig. 4.

Two phylogenetic clades showed an enrichment of BRENDA-EC161 functions. The first clade was made up by a group of 5 MAGs all belonging to the order *Microtrichales* (phylum *Actinobacteriota*). The second clade consisted of nearly half of the total number of MAGs. This large clade comprised several orders all belonging to the phylum *Proteobacteria*. Within this clade, high-gene density was notable in UBA10353, *Granulosicoccales*, *Pseudomonadales*, *Enterobacterales*, *Geminicoccales*, *Caulobacterales*, *Rhodobacterales*, and *Rhizobiales* orders. Conversely, a marked lower gene density was observed in the orders *Flavobacteriales*, *Chitinophagales*, and *Cytophagales* of the phylum *Bacteroidota*. All MAGs contained annotated BRENDA target functions except for *Sc*MAG22 and *Sc*MAG25, which were assigned to the orders *Flavobacteriales* and BD1-5, respectively (Additional file 1: Table S9).

The largest number of annotated genes (70) was found in the medium-quality *At*MAG62, assigned to *Granulosicoccales* which also revealed the largest number of target functions (30). Additionally, among the high-quality MAGs, the one with a substantial number of annotated target genes was *Sc*MAG11, belonging to *Pseudomonadales*, with 51 genes (Additional file 1: Table S9).

Focusing on BRENDA-EC42 functions, the genes encoding for putative halogenases revealed a higher density in the orders UBA10353, *Enterobacterales*, and *Caulobacterales* compared to other lineages (Fig. 4). In particular, the halogenases stand out in the *Caulobacterales*. Regarding genes encoding putative hydrolytic dehalogenases, they were broadly distributed along the entire tree, with a particularly notable presence in the *Microtrichales* clade, the UBA9160 order (phylum *Myxococcota*), and in certain *Proteobacteria* clades like UBA10353, *Pseudomonadales*, *Caulobacterales*, and *Rhizobiales*. Genes encoding non-hydrolytic functions were restricted to some lineages, e.g., *Granulosicoccales*, *Enterobacterales*, *Caulobacterales*, and *Rhizobiales*.

Among the 98 MAGs, the most frequently annotated BRENDA-EC42 function was the haloalkane dehalogenase (Additional file 1: Table S9). Specifically, this function was particularly annotated among the *Sc* MAGs, with as many as 10 genes observed in the *Pseudomonadales* MAG11.

### Biodegradative and biosynthetic potential of BRENDA-EC161 functions encoded by each MAG and taxonomic affiliations

**Fig. 5:**
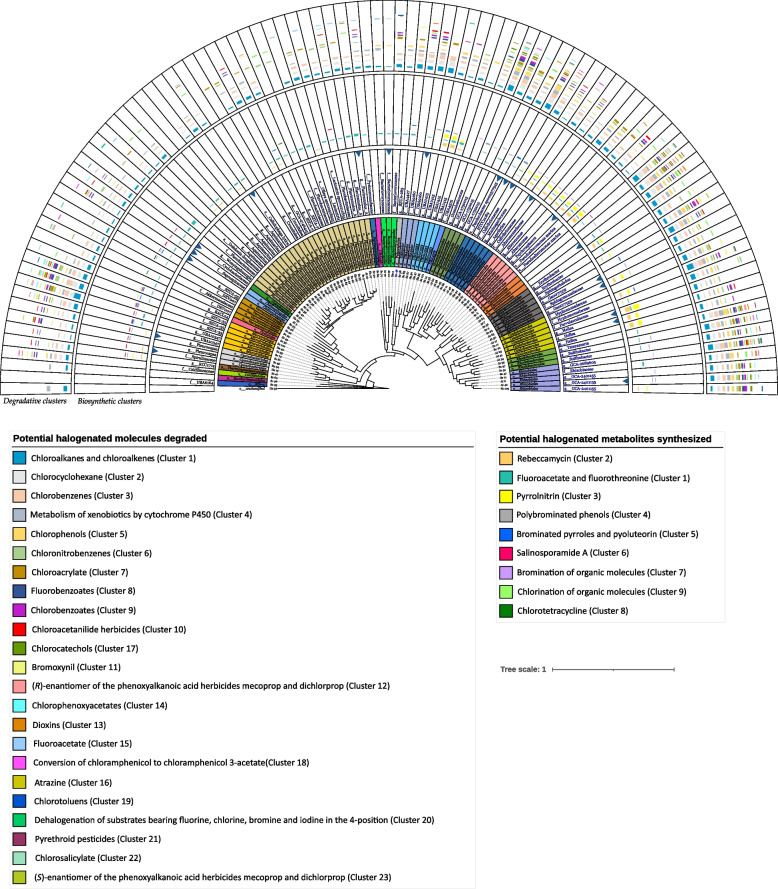
Phylogenetic tree showing for each MAG the lowest taxon assigned by GTDB-Tk and the clustered target genes according to the potential type of degraded or synthesized halogenated molecule. From inner to outside: MAGs numbered and macroalga host, colored taxonomic orders, lowest taxon assigned by GTDB-Tk (empty box indicates a novel family), MAG quality (the arrow indicates high-quality MAG), and the bar chart representing cluster density (with synthesized compounds in the inner circle and degraded compounds in the outer circle) for each MAG, aligned accordingly. The names of the compounds are listed in descending order of the detected cluster density values across all MAGs. Ref* indicates an unsupervised reference genome of the public KBase genomes database used in phylogenetic tree building. *Sc*, *Sphaerococcus coronopifolius*; *At*, *Asparagopsis taxiformis*; *Hs*, *Halopteris scoparia*

To establish the types of organo-halogenated molecules potentially metabolized by each MAG (biodegradation or biosynthetic potential), we applied the same approach adopted for the metagenomes. In this process, the annotated target genes of each MAG were clustered based on the common category of degraded molecules or metabolites produced by the BRENDA-EC161 enzymes they encoded. Subsequently, the gene density within the single clusters was calculated for each MAG (Additional file 1: Table S8). Furthermore, the phylogenetic tree involving the 98 MAGs was integrated with the densities of both biodegradative and biosynthetic clusters associated with each MAG. This integration enabled to cross-link these clusters to specific prokaryotic taxa (Fig. 5).

All 32 types of clusters identified within the metagenomes were also found within the MAGs. Regarding their potential ability to degrade halogenated molecules, MAGs assigned to orders such as *Microtrichales*, *Granulosicoccales*, *Pseudomonadales*, *Geminicoccales*, *Sphingomonadales*, *Rhodobacterales*, and *Rhizobiales* displayed similar profiles. Generally, these orders turned out to be those with the highest cluster density and a notable number of degradation clusters, reaching up to 18–20 per MAG (e.g., *Sc*MAG6 and *Sc*MAG47).

Genes encoding enzymes involved in aliphatic catabolism (clusters 1 and 2) were found in almost all MAGs with a more marked gene density in *Caulobacterales Sc*MAG17 and *Microtrichales Sc*MAG74.

Clusters associated with the degradation of aromatic compounds were spread along the phylogenetic tree showing specialization for specific halogenated molecules: *Microtrichales Sc*MAG53, *Pseudomonadales Sc*MAG11, *Rhizobiales Sc*MAG47, and UBA10353 *Sc*MAG50 for chlorobenzenes (cluster 3); *Rhodobacterales At*MAG35 for chlorophenols (cluster 5); *Microtrichales Sc*MAG53 for chloronitrobenzenes (cluster 6) and *Rhizobiales Sc*MAG109 for fluorobenzoates (cluster 8); *Granulosicoccales At*MAG62 and *Sphingomonadales Sc*MAG9 for chlorobenzoates (cluster 9); *Enterobacterales At*MAG53 for the chlorocatechols (cluster 17); and the *Geminicoccales At*MAG44 for the chlorotoluenes (cluster 19).

Some MAGs contained genes annotated for herbicide degradation. The *Microtrichales Sc*MAG53 and the *Pseudomonadales Sc*MAG11 were characterized by degradation clusters targeting phenoxyalkanoic acid herbicides in both *R*-enantiomer (cluster 12) and *S*-enantiomer (cluster 23). These two clusters were detected together within another *Microtrichales* MAG (*Sc*MAG74). Noteworthy, the *S*-enantiomer cluster alone was annotated in very few MAGs, almost all belonging to *Microtrichales* whereas the *R*-enantiomer cluster was only observed in some MAGs belonging to 9 orders, especially in *Microtrichales*. The chloroacetanilide cluster (10) was particularly detected in *Geminicoccales* and *Sphyngomonadales* MAGs, the bromoxynil cluster (11) within *Granulosicoccales* and *Rhizobiales* while the chlorophenoxyacetate cluster (14) in *Geminicoccales*, *Granulosicoccales*, and *Microtrichales*. Lastly, the atrazine cluster (16) was shared among 8 orders. No MAGs containing all the 6 herbicide clusters were found, but *Microtrichales Sc*MAG53 and *Pseudomonadales Sc*MAG11 showed 5 out of 6 clusters.

Degradation of xenobiotics by P450 enzymes (cluster 4) was more common in *Granulosicoccales*, *Geminicoccales*, *Caulobacterales*, *Sphingomonadales*, *Rhodobacterales*, and *Rhizobiales* particularly within the *Rhodobacterales At*MAG35. The chloroacrylate cluster (7) was also shared among different orders and the *Microtrichales Sc*MAG74 showed the higher density. The dioxins degradation cluster (13) was mainly observed in *Microtrichales* MAGs, followed by the *Granulosicoccales At*MAG62. As for the fluoroacetate (15), pyrethroid (21), chlorosalicylate (22), and chloramphenicol (18) clusters, the first 3 were observed in *Geminicoccales* (e.g., *Sc*MAG6) and *Rhizobiales* (e.g., *Sc*MAG47) whereas the latter was notably detected in *Bacteriovoracales* (e.g., *At*MAG70) and *Flavobacteriales* (e.g., *Sc*MAG33 and *Sc*MAG38). The dehalogenation cluster responsible for substrates containing fluorine, chlorine, bromine, and iodine in the 4-position (20) was observed only in 8 MAGs and the *Bacteriovoracales Hs*MAG5 and the *Rhizobiales Sc*MAG109 displayed a higher density.

As for the biosynthesis clusters, particularly those related to rebeccamycin (2) and pyrrolnitrin (3) were significantly observed in UBA10353, *Pseudomonadales*, *Caulobacterales*, and *Enterobacterales MAGs*, with a high density within the *Caulobacterales* (e.g., *At*MAG29) (Fig. 5). The cluster related to the chlortetracycline biosynthesis (8) was mainly associated with the *Verrucomicrobiales* MAGs. In contrast, the fluoroacetate and fluorothreonin cluster (1) showed a large distribution across the tree, with a density range from 0.1 (*Granulosicoccales At*MAG62) to 1.7 (*Flavobacteriales Sc*MAG42). Conversely, the salinosporamide A cluster (6) was observed in some orders on the left side of the tree.

The cluster of brominated pyrroles and pyoluteorin (5) was mostly observed in *Microtrichales* among few others. The polybrominated phenols cluster (4) was recorded in different orders, particularly in all *Enterobacterales* MAGs. Noteworthy, among the numerous *Flavobacteriales* this cluster was less represented as compared to the clusters of bromination (7) and chlorination (9) of organic molecules. The same was observed for *Caulobacterales* MAGs. Clusters 7 and 9 were not widespread across the obtained MAGs. Chlorination was detected in only 4 *Microtrichales* MAGs and 1 *Sphingomonadales* MAG (AtMAG30). Interestingly, the *Microtrichales Sc*MAG53 showed all 3 clusters (4, 7, and 9).

## Discussion

In red and brown algae, a rapid emission of iodinated halocarbons and other halometabolites has been particularly observed during the induction of oxidative burst [46, 47]. The genetic and biochemical reconstitution of bromoform biosynthesis in *At* has suggested that bromoform and other halogenated molecules found in marine algae are produced as a part of a cascade involved in the production and manipulation of reactive oxygen species. This cascade converts hydrogen peroxide into defensive halogenated metabolites [48]. Some of these halogenated metabolites have been suggested to possess antibacterial properties against marine prokaryotes, potentially serving as a defense mechanism in certain macroalgal taxa, including the *Asparagopsis* genus [18, 49–51]. However, this marine macroalgal defense mechanism might reduce the microbial epibiosis, potentially eliminating bacteria crucial for extending the algae’s defense mechanisms. Despite this, there has been no in-depth investigation into the genetic content of prokaryotic fractions within algal microbiomes that might be actively involved in controlling the fate of host-secreted halocompounds. This study presents the first in-depth shotgun-metagenomics analysis on macroalga-associated microbes, focusing on their gene repertoires involved in degrading or transforming halogenated metabolites. The study specifically examines 3 sympatric Atlantic macroalgae: two red, *Sphaerococcus coronopifolius* (*Sc*) and *Asparagopsis taxiformis* (*At*), and one brown, the *Halopteris scoparia* (*Hs*). In our work, we investigated *At* due to its broad and prolific biosynthesis of halometabolites [51–53], its widespread distribution in warm-temperate, tropical, and subtropical waters [54], and the availability of its genomic sequence [48]. *At* is also a focus of significant research, particularly in innovative biological feed studies for methane emission reduction in cattle due to its halometabolite content, especially bromoform [55].

To understand whether a similar halo-metabolic landscape is shared in seaweeds, we also collected two other sympatric macroalgae, one red (*Sc*) and one brown (*Hs*), inhabiting the same sampling area under similar local conditions of pressure and sharing the same microbial community seeded by seawater.

### *Sc*,* At*, and *Hs* are distinct ecological niches, each harboring a unique microbiome

In line with previous studies on macroalgae [56], our survey based on the 16S rRNA gene has revealed a wide range of prokaryotic phyla associated with *At*, *Sc*, and *Hs* macroalgae (Fig. 1). The taxonomic annotation of MAGs has confirmed the presence of several bacterial phyla, including the parasites *Patescibacteria* and *Bdellovibrionota*. Additionally, in the *Hs* microbiome, we identified a unique Archaea phylum, the ammonium oxidizing *Crenarchaeota* (also known as *Thermoproteota*), previously found to be associated with some macroalgal hosts [57–59]. This finding suggests a predominant presence of the bacterial fraction in macroalgae-associated communities. Furthermore, as formerly observed in other macroalgae microbiomes [57], the 3 algae shared almost all the observed phyla and classes (e.g., *Proteobacteria*, *Gamma*- and *Alphaproteobacteria*, *Bacteroidia*, *Planctomycetes*, *Actinobacteria*, *Verrucomicrobia*) while a diversification was noticed at lowest phylogenetic ranks. A low number of shared taxa (17.5%) was observed among the three macroalgae, when only known classified genera were considered (Additional file 2: Fig. S2), suggesting that, despite being exposed to the same seawater community and environmental drivers, they represent distinct ecological niches able to select specific prokaryotic communities. Further, the overlap in taxonomic composition between the red algae (*Sc* and *At*) did not show a wider similarity than between the red-brown algae (*Sc*-*Hs* and *At*-*Hs*).

Only *At* microbiome has been previously described [60], but our observations did not reveal a complete match (data not shown). Rather, we found that some bacterial orders previously observed separately in the coastal continental and island *At* samples were both present in our coastal continental *At* sample. Both coastal and mainland *At* samples (here and previous work) were nevertheless collected in the same marine area but in different seasons. Overall, these taxonomic data suggest that the shaping of the microbiota in the lower taxonomic levels is not strictly dependent on the taxonomy of the alga, at species or division level.

### Macroalgae metagenomes and MAGs reveal a versatile organohalogen degradative and biosynthetic potential involving previously unknown taxa

Despite investigations into the ability of individual marine bacteria or phyla to degrade or synthesize halogenated molecules [61–63], the role of the entire marine microbial community in the management of these compounds remains unknown. Our examination of each macroalga metagenome revealed a consistent potential for organohalogen degradation, surpassing the biosynthetic one. This suggests that the microbes associated with these three algae possess a specialization primarily in degrading halogenated molecules rather than synthesizing them. Interestingly, while the three algae showed a wide diversity in the phylogenetic composition of the associated microbiomes, they basically shared all common categories of degraded molecules or produced metabolites (Fig. 3). A priori one might assume that the assembly of microbial BRENDA-EC161 functions on algae is not based on the selection of specific prokaryotic ranks. The lottery hypothesis stating that bacterial communities assemble based on functional genes rather than species was first introduced through research conducted on the green alga *Ulva australis* [64]. However, the research performed in our current study, examining BRENDA-EC161 functions within MAGs and their taxonomic affiliations, showed that these functions are primarily clustered within specific bacterial ranks. A novel ecological and genetic finding is the potential involvement of uncultured members from relatively unknown prokaryotic orders, such as *Caulobacterales*, *Granulosicoccales*, *Geminicoccales*, UBA10353, UBA9160, *Microtrichales* in organohalogen metabolism (Fig. 4). Additionally, newly identified uncultured representatives from more recognized bacterial orders (e.g., *Rhizobiales*, *Rhodobacterales*, *Sphingomonadales, Enterobacterales, Pseudomonadales*) are implicated in this process as well.

A specific richness in BRENDA-EC161 degradative functions was uncovered in the macroalga-associated MAGs assigned to the orders *Rhizobiales*, *Rhodobacterales*, *Caulobacterales*, *Sphingomonadales*, *Geminicoccales*, *Pseudomonadales*, *Granulosicoccales*, and *Microtrichales* (Fig. 4). Considering the entire community, from the taxonomic profile based on 16S rRNA gene detected for the *Sc*, *At*, and *Hs* metagenomes, these orders were particularly present in *Sc*, followed by *At* and *Hs* (Additional file 2: Fig. S2). This aspect seems to be in line with the organohalogen degradation cluster density values detected in the metagenomes (Fig. 3A). Basically, an overall trend is observed in the density values, with *Sc* exhibiting higher densities than *At*, which in turn are higher than those of *Hs*. We hypothesize that the higher gene density values of the BRENDA-EC161 degradative clusters observed for the two red algae metagenomes, especially in *Sc*, might be related to a greater secretion of halometabolites compared to the brown alga. Likely, this could justify the richer microbiome in *Sc*, followed by *At*, with bacterial ranks more enriched in BRENDA-EC161 functions that degrade organohalogens. Accordingly, higher levels of halogenated secondary metabolite production in red algae, compared to all macroalgae, have been documented [65, 66].

Regarding the spectrum of organohalogen biodegradative functions, our analysis revealed a preeminent set of genes encoding putative hydrolytic dehalogenases (EC 3.8.1.X) (BRENDA-EC42 subgroup) in both metagenomes (Fig. 2) and MAGs (Fig. 4 and Additional file 1: Table S9). This was particularly evident in clusters associated with degradation reactions of haloalkanes and haloalkenes (Fig. 3A). Notably, among these, the haloalkane dehalogenases (EC 3.8.1.5) displayed the highest density values, following a trend *Sc* > *At* > *Hs* (Fig. 2). The putative hydrolytic dehalogenases encoded by the assembled MAGs are known to convert a wide range of halocarbons, including halogenated alkanes, cycloalkanes, alkenes, ethers, alcohols, ketones, cyclic dienes, alkanoic acids, and acetates [67, 68]. Genes encoding these BRENDA-EC42 functions were particularly abundant in MAGs corresponding with *Microtrichales*, UBA9160, UBA10353, *Pseudomonadales*, *Caulobacterales*, and *Rhizobiales* (Fig. 4). Several are the macroalga species for which the ability to secrete a multitude of halocarbons was demonstrated, at various latitudes [65, 66, 69]. Specifically, *At* is a prolific producer of these compounds, especially (mono or poly) iodinated, brominated, and/or chlorinated methanes (particularly bromoform), ethanes, propanes, epoxy-propanes, ethanols, isopropanols, ethylenes, propenes, butenols, acetones, acetates, acetic, and acrylic acids [52, 65, 66]. Notably, *At* is a haloacrylic acid producer [53], which seems to align with our discovery of a gene cluster involved in chloroacrylate degradation (Fig. 3A). The most annotated genes within this cluster encode for the 2-haloacrylate reductase (EC 1.3.1.103) and the (*S*)-2-haloacid dehalogenase (EC 3.8.1.2) (Fig. 2). In the soil bacterium *Burkholderia* sp. WS, these enzymes constitute a probable pathway for metabolizing 2-chloroacrylate into (*R*)-lactate [70]. The 2-haloacrylate reductase catalyzes the conversion of 2-chloroacrylate into (*S*)-2-chloropropionate, which is subsequently dehalogenated by (*S*)-2-haloacid dehalogenase into (*R*)-lactate [70]. Genes encoding both enzymes were found in several of the assembled MAGs of *At* and *Sc*, particularly in those assigned to *Granulosicoccales*, *Geminicoccales*, and *Rhizobiales* (Fig. 5 and Additional file 1: Table S9).

Concerning *Sc* and *Hs*, we did not find exhaustive studies reporting the characterization of the secreted organic halogenated fraction, especially halocarbons. Nevertheless, the prevalence of hydrolytic dehalogenases among the annotated BRENDA-EC161 functions in the distinct *Sc*, *At*, and *Hs* metagenomes studied here suggests that hydrolytic dehalogenation could be a common trait among coastal algal microbiomes related to the secretion host halocarbons.

Our analysis also identified gene clusters potentially involved in the breakdown of various organic halogen compounds containing the benzene ring (Fig. 3A). Previous research highlighted the ability of macroalgae to produce haloaryl metabolites, such as halophenols [65, 71]. Despite studies demonstrating the production of phenolic compounds in *At* extract, the precise chemical structures of these metabolites remain elusive [72]. However, two bromophenols have recently been identified in *At* [73]. In the present study, genes encoding glutathionyl-hydroquinone reductases (EC 1.8.5.7) (Fig. 2), a recently identified subset of glutathione transferases that catalyze the GSH-dependent reduction of glutathionyl-hydroquinones conjugates to hydroquinones, resulted particularly annotated in the cluster of genes degrading halophenols (Fig. 3A). In bacteria these enzymes also catalyze specific reduction of *S*-glutathionyl-(chloro)hydroquinone [74–76]. Moreover, we observed gene clusters potentially active on anthropogenic haloaromatic molecules (pollutants, herbicides, and pesticides) revealing a multiform degradative potential of organohalogens. Previous biosorption studies showed the interaction between macroalga biomass and aromatic contaminants like toluene, benzene, and polycyclic molecules [77, 78]. Furthermore, in controlled environments like photobioreactors, there have been recent examples of symbiotic relationships forming between bacteria and unicellular algae. These bacteria possess the ability of controlling the fate of aromatic anthropogenic pollutants [79, 80].

Noteworthy, the analysis of three metagenomes revealed a high-density cluster of genes encoding putative cytochrome P450 enzymes as well as glutathione transferases, able to degrade halogenated molecules. The P450 enzymes’ ability to bind foreign liposoluble halogenated hydrocarbons was among the first biochemical studies of these proteins [81]. These enzymes are present in both prokaryotes and eukaryotes and are known for metabolizing xenobiotics, where hydroxylation converts insoluble hydrocarbons into more soluble for easier elimination [82]. A variety of halogenated xenobiotics (e.g., therapeutic agents and agrochemicals) are bound by the lipophilic P450 active site to be detoxified through a dehalogenation step [83–85]. Prokaryotic glutathione transferases are key enzymes in the cellular detoxification of a broad range of harmful xenobiotics, including aliphatic, aromatic, and heterocyclic molecules with halide groups [86]. These enzymes catalyze glutathione conjugation to electrophilic groups (mainly introduced by P450) of a wide range of hydrophobic toxic compounds, thus promoting their excretion from the cell [87]. Glutathione transferases detoxify several classes of herbicides including triazines, a class of man-made chemicals that includes atrazine, one of the most widely used chlorinated herbicides [86].

Collectively, the genes encoding P450 enzymes, glutathione transferases, hydrolytic dehalogenases, and other brenda-EC161 functions annotated here may be a bacterial integrated system useful in controlling the impact of synthetic, natural, and host-secreted organohalogens on the microbial community. While genes encoding P450 enzymes and glutathione transferases are mostly ubiquitous across the assembled MAGs, they are particularly abundant in those assigned to the *Proteobacteria* phylum, particularly in *Rhizobiales*, *Rhodobacterales*, *Sphingomonadales*, *Geminicoccales*, and *Granulosicoccales* (Fig. 5).

Considering the potential synthesized metabolites by the three algal metagenomes (Fig. 3B), a broader trend was seen for the biosynthesis of antimicrobial compounds (fluoroacetate, fluorothreonine, rebeccamycin, pyoluteorin, pyrrolnitrin, salinosporamide A, chlortetracycline), potentially serving as drivers influencing microbiome assembly. There was a marked heterogeneity in the biosynthetic properties of *Sc*, *At*, and *Hs* metagenomes. Although *Sc* metagenome encodes a higher number of BRENDA-EC161 functions than *At* and *Hs*, its biosynthetic clusters tend to have lower gene density values, particularly with those of rebeccamycin and pyrrolnitrin. This data was confirmed by the biosynthetic gene clustering of MAGs (Fig. 5), where *At* MAGs (e.g., *Enterobacterales*, *Sphingomonadales*, and *Caulobacterales*) were particularly enriched in genes involved in the biosynthesis of these two antimicrobials. The variability in gene density values within biosynthetic clusters among the three metagenomes suggests that the potential halometabolites production in algal microbiomes likely depends on intrinsic properties of individual holobionts. We hypothesize that in the alga-microbiota symbiotic relationship, the lower ability of the host-microbe to produce halometabolites might correspond to a greater algal ability to synthesize them. However, a heterogeneous bacterial synthesis of halometabolites, in particular antimicrobials (e.g., rebeccamycin and pyrrolnitrin), may contribute along with the algal production of halometabolites to select, assemble, and maintain a distinct algal microbiota characterized by unique species diversity.

### Organohalogen metabolism genes: microbiome assembly guided by the holobiont as a defense mechanism against environmental stresses

Investigations into the distribution of specific xenobiotics (pharmaceuticals), herbicides, and pesticides in estuarine and seawater along the Portuguese coast, including sampling sites close to the harbor bay of Lagosteiros, have reported the presence of halogenated chemicals, particularly atrazine and alachlor (a chloroacetanilide) herbicides [88]. Recently, contamination by atrazine and alachlor as well as pesticides such as chlorinated pyrethroids and hexachlorocyclohexane isomers has been detected across seasons in water and sediments sampled from the estuaries of Tagus and Douro Rivers (NE Atlantic Ocean Portuguese coast), while searching for thirty-seven endocrine disruptor compounds [89]. The atrazine and alachlor negative effect on the growth of microalgae in vitro has been reported [90].

We suspect that the identified gene clusters within the here analyzed metagenomes and associated MAGs, potentially involved in the metabolism of anthropogenic organohalogens such as chlorocyclohexane, chlorobenzenes, chlorophenols, xenobiotics, pesticides (e.g., pyrethroids), and herbicides (e.g., chlorocetanilides and atrazine) (Figs. 3A and 5), could represent an adaptative mechanism of the coastal *Sc*, *At*, and *Hs*, intermittently exposed to anthropic perturbations. The bacterial ability to biodegrade halogenated herbicides such as atrazine and chlorocetanilides originating from agricultural runoff into coastal waters may therefore preserve the macroalgal fitness ensuring the survival of the entire holobiont. Our hypothesis is that the macroalgal secretion of halometabolites could be evolutionary linked with the biogenic and abiogenic naturally occurring organohalogen compounds in the marine environment. The secretion drives the selection of a degrading microbial community recruited to control the natural halomolecules with a phytotoxic activity and, in the anthropization era, those of anthropic origin having structural similarity. A costal holobiont active in the metabolism of organohalogen compounds would have formed and evolved over time. The macroalgal holobiont appears to favor bacterial populations encoding enzymes with a broad substrate promiscuity, capable of metabolizing both naturally occurring exudates and structurally similar pollutants, thereby establishing a beneficial cycle for the environment. Marine bacteria with haloalkane dehalogenases showing an exceptionally broad substrate specificity have been previously described [91, 92].

An increased rate of halocarbon secretion by red polar marine macroalgae due to stresses induced by altered environmental conditions has been demonstrated, suggesting that global warming and uncontrolled ocean eutrophication may have a significant role in algal halometabolites secretion [93]. In plants, the so-called “cry-for-help” has been described as a mechanism driven by the modification of root exudate chemistry induced by pathogens and predators, aimed to recruit a beneficial microbiome exerting a plant protection effect. The same mechanism has been recently proposed to occur in plants growing in polychlorobiphenyl contaminated soils, in which the root-exudation-mediated microbial recruitment enriches in the plant rhizosphere a complex metabolic network active in contaminant degradation, in turn allowing the plant to rapidly adapt to the phytotoxic stress conditions [94]. It cannot be excluded that the “cry-for-help” mechanism observed in plants under stress conditions, could also be employed in macroalgae. We hypothesize that organohalogen production in macroalgae, besides defending against pathogens, foulers, and herbivores, might act as an ecological driver for recruiting beneficial microbes into the holobiont assembly, thereby reducing the chemical environmental phytotoxicity.

### Genetic potential for bioprospecting

Hydrolytic dehalogenases, a prominent gene group among the annotated BRENDA-EC42 functions, play a crucial role in economical and environmentally friendly industrial processes and biotechnological applications [68, 95–97]. While extensively studied in soil bacteria commonly exposed to manufactured organohalogens (e.g., herbicides and pesticides) [98], recent marine genomics and metagenomics investigations have revealed microbial hydrolytic dehalogenases with unique functional and structural properties [91, 92, 99]. Lately, the marine haloalkane dehalogenase DmmA, encoded by a metagenomic DNA fragment, and exhibited exceptional substrate specificity in degrading several environmental pollutants that are resistant to other closely related enzymes [100].

By studying specific marine holobionts, we have unveiled a significant blue genetic potential, including putative bacterial haloalkane dehalogenases, among other dehalogenases and halogenases. These discoveries provide a highly helpful resource for future experimental verification. Furthermore, many genes were assigned to different uncultured taxa based on MAG taxonomic classification. The microbial genetic diversity, particularly from novel or poorly known taxa, is currently an added value for the development of synthetic biology to design specific heterologous recombinant DNA circuits to be expressed in engineered microbial platforms and address application challenges outside-the-lab such as bioremediation and bio-based synthesis of chemical building blocks [27, 101]. Expression of heterologous genes in engineered microbial platforms could offer novel solutions to the mineralization of recalcitrant contaminants [102] or organohalide production [26]. In addition, genes encoding halogenases, such as the tryptophan halogenases, are today an emerging opportunity in the integration of synthetic biology and synthetic chemistry (GenoChemetics). This integration allows the fine-tuning of the bioactivity, bioavailability, and reactivity of medicinally and agriculturally relevant aromatic and aliphatic compounds via selective C-H functionalization [25]. This expansion enhances the portfolio of commercially important organohalides [103, 104].

## Conclusion

The comprehensive data presented here enriches our understanding on the composition and functions of the macroalgae-associated bacterial community. This work helps to unravel the still largely unknown role of the microbial dark matter. The analysis of specific MAGs encoded functions provides insights into their potential involvement in organohalogen metabolism, offering the possibility to develop innovative halo- and dehalogenation biocatalysts.

### Supplementary Information

**Additional file 1: Table S1.** *Sphaerococcus coronopifolius*. **Table S2.** Sequencing Metadata. **Table S3. ***Sphaerococcus coronopifolius*. **Table S4.** High- and medium-quality MAGs. Genome sizes, completness and contaminations estimated are reported. **Table S5.** GTDB-Tk taxonomic classification of *Sphaerococcus coronopifolius* MAGs. **Table S6.** Number of novel candidate taxa emerged after the GTDB-Tk classification of binned prokaryotic MAGs. **Table S7.** Gene density. **Table S9.** Annotated BRENDA-EC161 functions* in each single MAG§ and taxonomic affiliation^†^.

**Additional file 2: Fig. S1.** Venn diagram based on the number of prokaryotic orders assigned to metagenomic contigs (SSU rRNAs taxonomic analysis). *Sc*, *Sphaerococcus coronopifolius*; *At*, *Asparagopsis taxiformis* and *Hs*, *Halopteris scoparia*. **Fig. S2.** Classified prokaryotic genera of macroalgal-associated microbiomes based on SSU rRNAs analysis. A, Venn diagram reporting the numbers of shared and unique genera. B, List of shared and unique prokaryotic genera of the 3 macroalgal microbiomes and related taxonomic orders. Orders also assigned to MAGs are in bold. MAGs orders enriched in BRENDA-EC161 functions are highlighted in yellow. *Sc*, *Sphaerococcus coronopifolius*; *At*, *Asparagopsis taxiformis* and *Hs*, *Halopteris scoparia*. **Fig. S3.** Prokaryotic phyla assigned by GTDB-Tk to macroalgal MAGs. Number of MAGs assigned per Phylum are indicated in *Sc*, *At* and *Hs* columns. Percentage is also indicated (upper bar chart). *Sc*, *Sphaerococcus coronopifolius*; *At*, *Asparagopsis taxiformis* and *Hs*, *Halopteris scoparia*. **Fig. S4.** Prokaryotic classes assigned by GTDB-Tk to macroalgal MAGs. Number of MAGs assigned per class are indicated in *Sc*, *At* and *Hs* columns. Percentage is also indicated (upper bar chart). *Sc*, *Sphaerococcus coronopifolius*; *At*, *Asparagopsis taxiformis* and *Hs*, *Halopteris scoparia*. **Fig. S5.** Prokaryotic orders assigned by GTDB-Tk to macroalgal MAGs. Number of MAGs assigned per order are indicated in *Sc*, *At* and *Hs* columns. Percentage is also indicated (upper bar chart). *Sc*, *Sphaerococcus coronopifolius*; *At*, *Asparagopsis taxiformis* and *Hs*, *Halopteris scoparia*. **Fig. S6.** Phylogenetic analysis of *Hs*MAG32 by FastTree2. The phylogenetic tree based on alignment similarity of a set of 49 core, universal genes defined by COGs, was developed considering an unsupervised set of 20 closely related genomes available on the public KBase genomes database. *Hs*MAG32 highlighted in yellow is positioned in the Archaea domain. *Hs*, *Halopteris scoparia*. The local-bootstrap support values are indicated in red. **Table S1.** List of 161 complete EC numbers*, corresponding to as many enzymes^†^ characterized to be involved in the cellular metabolism of halogens, available in BRENDA (Braunschweig Enzyme Database). **Table S2.** BRENDA-EC161* and -EC42^‡^ functions annotated for each macroalgal metagenome.

## Data Availability

Raw sequencing data and metadata are available at the following link: https://dataview.ncbi.nlm.nih.gov/object/PRJNA915238?reviewer=v47i8nc2gqedcte9p627rqgl7a.
