# Supplementary material for: Macroalgal microbiomes unveil a valuable genetic resource for halogen metabolism
Source: Microbiome. 2024 Mar 7;12:47. doi: 10.1186/s40168-023-01740-6 (PMC10919026; doi:10.1186/s40168-023-01740-6)
Supplement: Supplementary file 3 — Additional file 2: Fig. S1. Venn diagram based on the number of prokaryotic orders assigned to metagenomic contigs (SSU rRNAs taxonomic analysis). Sc, Sphaerococcus coronopifolius; At, Asparagopsis taxiformis and Hs, Halopteris scoparia. Fig. S2. Classified prokaryotic genera of macroalgal-associated microbiomes based on SSU rRNAs analysis. A, Venn diagram reporting the numbers of shared and unique genera. B, List of shared and unique prokaryotic genera of the 3 macroalgal microbiomes and related taxonomic orders. Orders also assigned to MAGs are in bold. MAGs orders enriched in BRENDA-EC161 functions are highlighted in yellow. Sc, Sphaerococcus coronopifolius; At, Asparagopsis taxiformis and Hs, Halopteris scoparia. Fig. S3. Prokaryotic phyla assigned by GTDB-Tk to macroalgal MAGs. Number of MAGs assigned per Phylum are indicated in Sc, At and Hs columns. Percentage is also indicated (upper bar chart). Sc, Sphaerococcus coronopifolius; At, Asparagopsis taxiformis and Hs, Halopteris scoparia. Fig. S4. Prokaryotic classes assigned by GTDB-Tk to macroalgal MAGs. Number of MAGs assigned per class are indicated in Sc, At and Hs columns. Percentage is also indicated (upper bar chart). Sc, Sphaerococcus coronopifolius; At, Asparagopsis taxiformis and Hs, Halopteris scoparia. Fig. S5. Prokaryotic orders assigned by GTDB-Tk to macroalgal MAGs. Number of MAGs assigned per order are indicated in Sc, At and Hs columns. Percentage is also indicated (upper bar chart). Sc, Sphaerococcus coronopifolius; At, Asparagopsis taxiformis and Hs, Halopteris scoparia. Fig. S6. Phylogenetic analysis of HsMAG32 by FastTree2. The phylogenetic tree based on alignment similarity of a set of 49 core, universal genes defined by COGs, was developed considering an unsupervised set of 20 closely related genomes available on the public KBase genomes database. HsMAG32 highlighted in yellow is positioned in the Archaea domain. Hs, Halopteris scoparia. The local-bootstrap support values are indicated in [file 40168_2023_1740_MOESM2_ESM.docx]

**
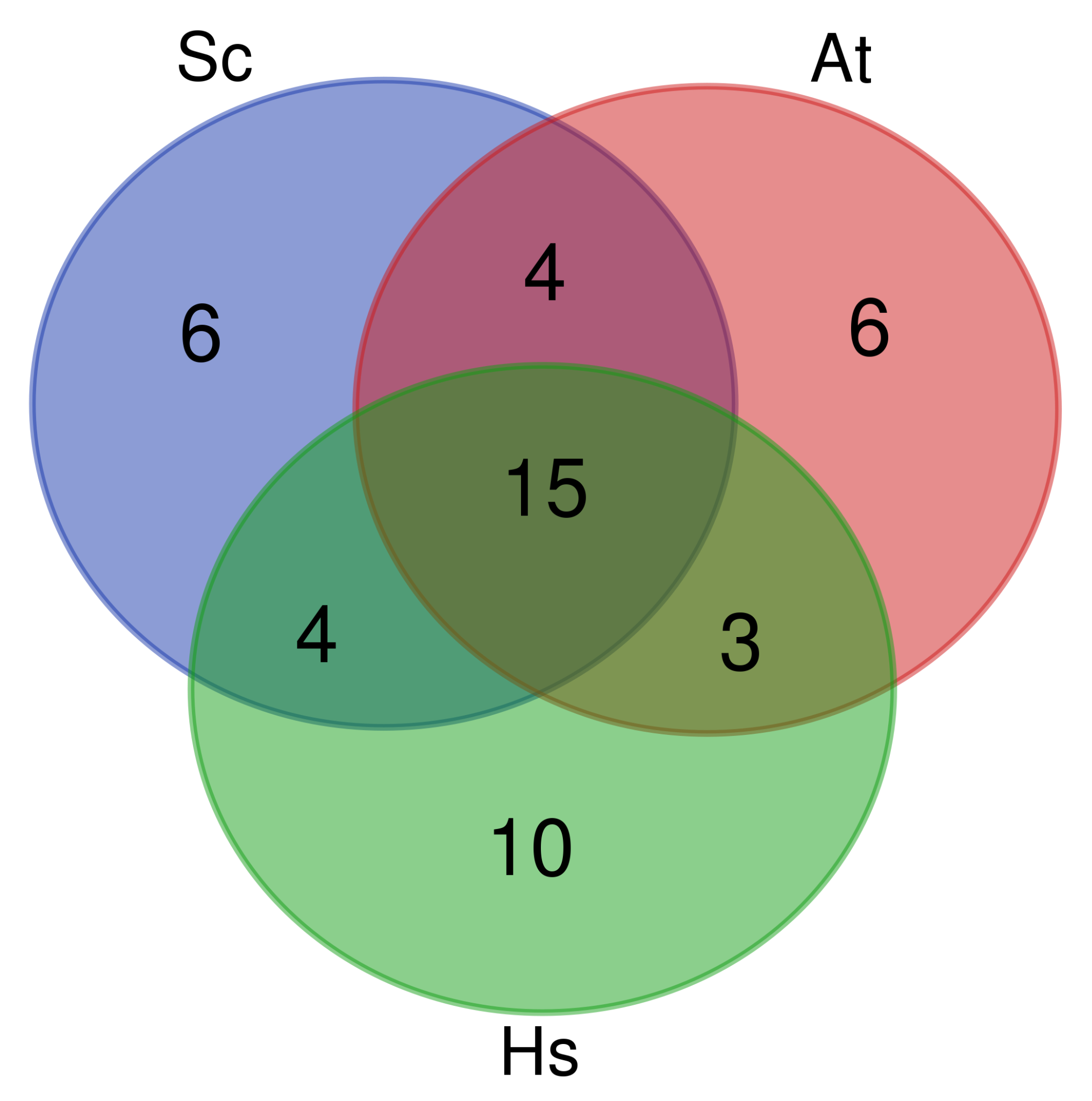
**

**Fig. S1** Venn diagram based on the number of prokaryotic orders assigned to metagenomic contigs (SSU rRNAs taxonomic analysis). *Sc*, *Sphaerococcus coronopifolius*; *At,* *Asparagopsis taxiformis* and *Hs, Halopteris scoparia*.

**
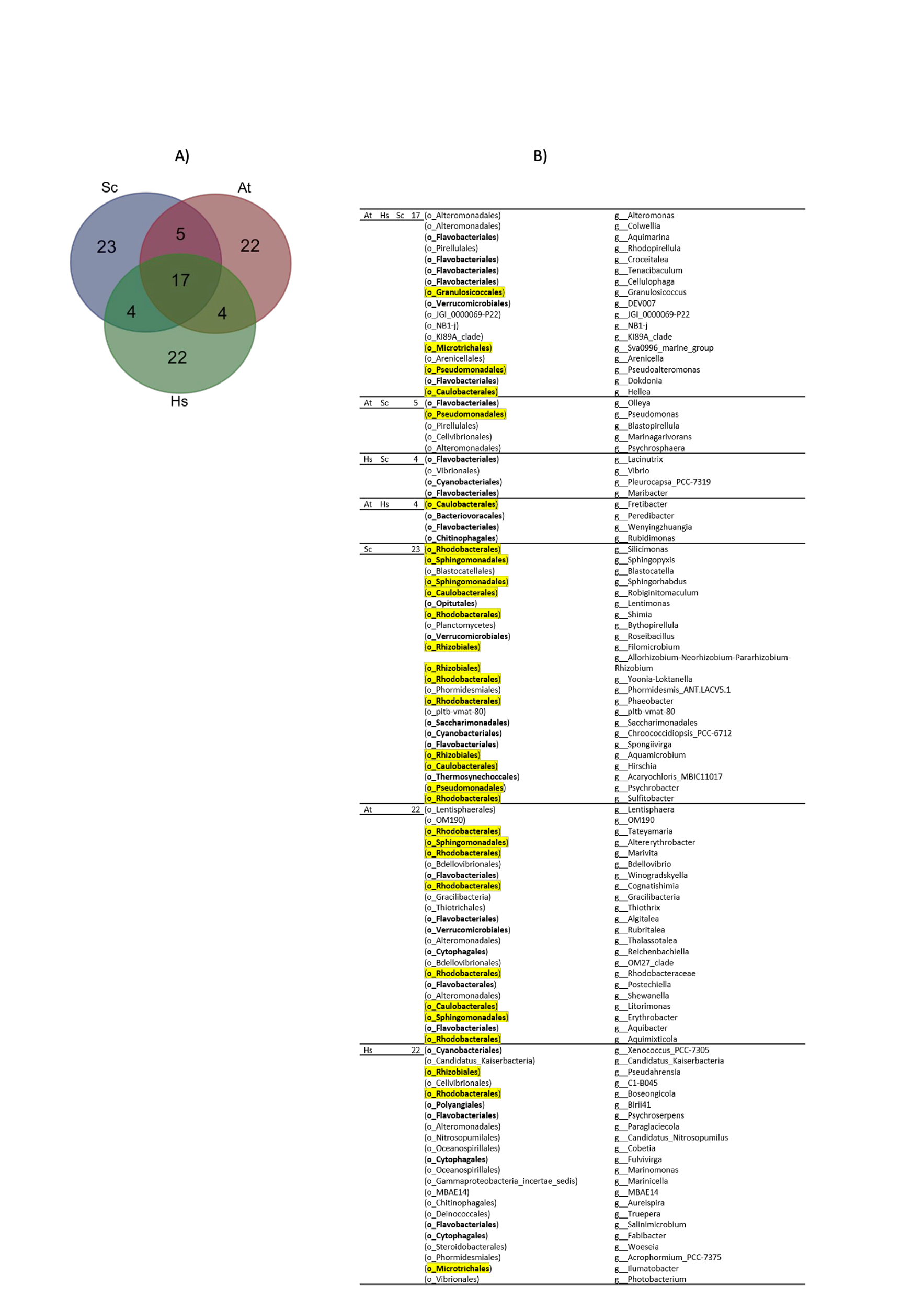
**

**Fig. S2** Classified prokaryotic genera of macroalgal-associated microbiomes based on SSU rRNAs analysis. **A**, Venn diagram reporting the numbers of shared and unique genera. **B**, List of shared and unique prokaryotic genera of the 3 macroalgal microbiomes and related taxonomic orders. Orders also assigned to MAGs are in bold. MAGs orders enriched in BRENDA-EC161 functions are highlighted in yellow. *Sc*, *Sphaerococcus coronopifolius*; *At,* *Asparagopsis taxiformis* and *Hs, Halopteris scoparia*.


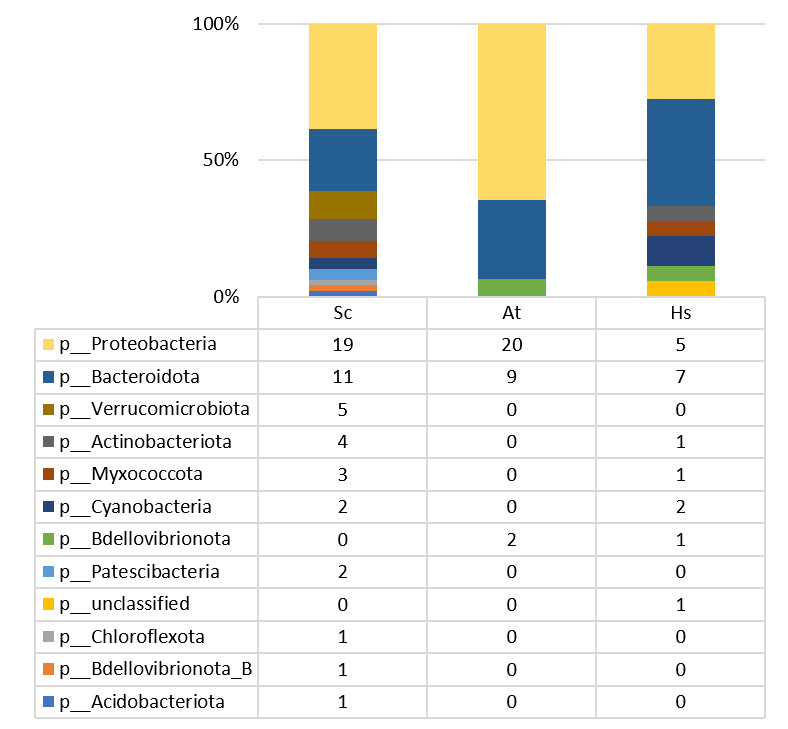


**Fig. S3** Prokaryotic phyla assigned by GTDB-Tk to macroalgal MAGs.  Number of MAGs assigned per Phylum are indicated in *Sc*, *At* and *Hs* columns.  Percentage is also indicated (upper bar chart).  *Sc*, Sphaerococcus *coronopifolius*; *At,* *Asparagopsis taxiformis* and *Hs, Halopteris scoparia*.


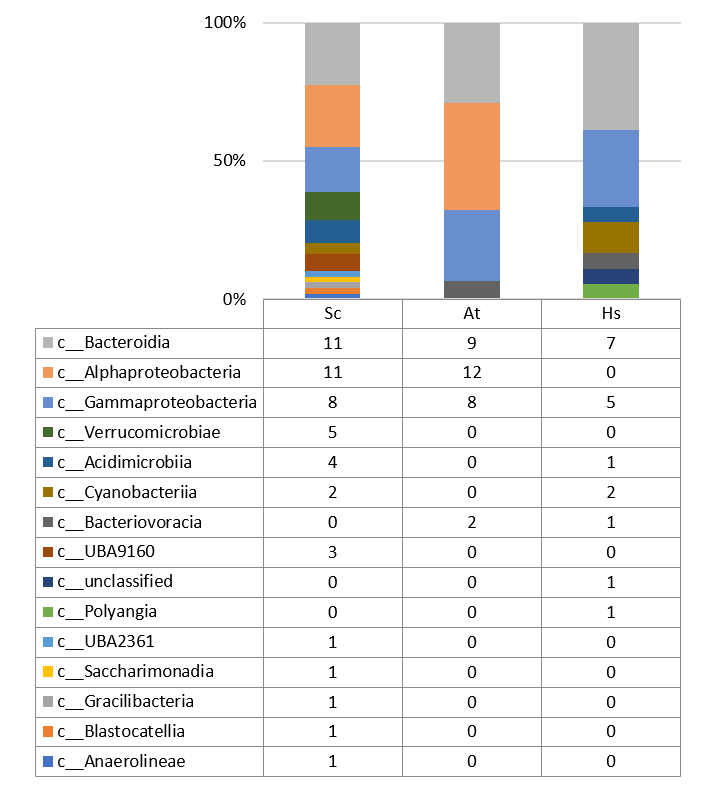


**Fig. S4** Prokaryotic classes assigned by GTDB-Tk to macroalgal MAGs. Number of MAGs assigned per class are indicated in *Sc*, *At* and *Hs* columns.  Percentage is also indicated (upper bar chart). *Sc*, *Sphaerococcus coronopifolius*; *At, Asparagopsis taxiformis* and *Hs, Halopteris scoparia*.


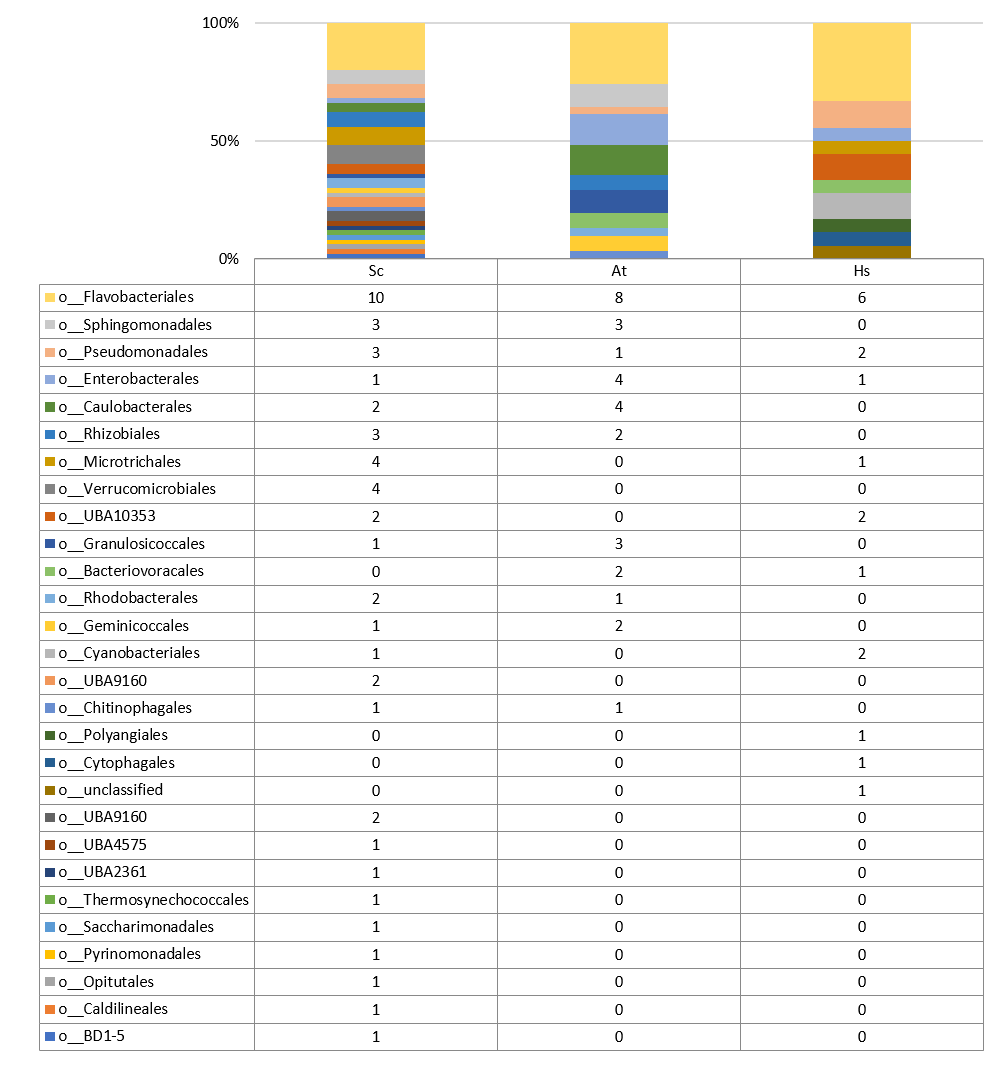


**Fig. S5** Prokaryotic orders assigned by GTDB-Tk to macroalgal MAGs. Number of MAGs assigned per order are indicated in *Sc*, *At* and *Hs* columns.  Percentage is also indicated (upper bar chart). *Sc*, *Sphaerococcus coronopifolius*; *At,* *Asparagopsis taxiformis* and *Hs, Halopteris scoparia*.

**
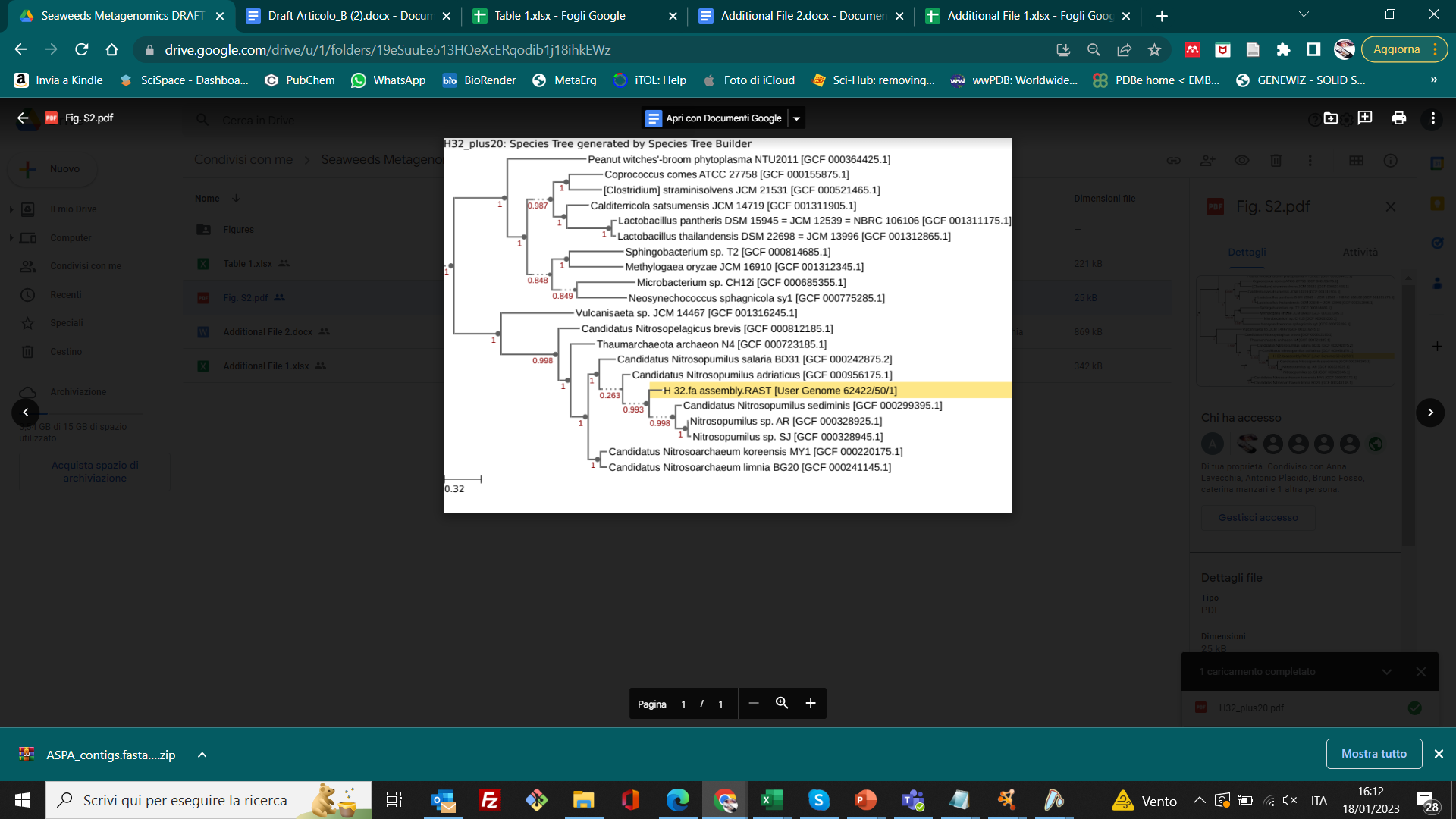
**

**Fig. S6** Phylogenetic analysis of *Hs*MAG32 by FastTree2. The phylogenetic tree based on alignment similarity of a set of 49 core, universal genes defined by COGs, was developed considering an unsupervised set of 20 closely related genomes available on the public KBase genomes database. *Hs*MAG32 highlighted in yellow is positioned in the Archaea domain. *Hs, Halopteris scoparia*. The local-bootstrap support values are indicated in red.

**Table S1** List of 161 complete EC numbers*****, corresponding to as many enzymes^†^ characterized to be involved in the cellular metabolism of halogens, available in BRENDA (Braunschweig Enzyme Database).

| **BRENDA EC number** | **BRENDA enzymatic activity** | **Pathway and functions detected from BRENDA Databases (IUBMB, KEGG, MetaCyc and UniProtKB comments are included when available)^§^** |
| --- | --- | --- |
| 1.1.1.1 | Alcohol dehydrogenase | KEGG: Chloroalkane and chloroalkene degradation, Metabolism of xenobiotics by cytochrome P450 (trichloroethylene degradation). |
| 1.1.1.225 | Chlordecone reductase | IUBMB comments: Chlordecone is an organochlorine pesticide. |
| 1.1.1.404 | Tetrachlorobenzoquinone reductase | IUBMB comments: Contains FMN. The enzyme, characterized from the bacterium *Sphingobium chlorophenolicum*, participates in the degradation of pentachlorophenol. KEGG: Chlorocyclohexane and chlorobenzene degradation. MetaCyc: Pentachlorophenol degradation. |
| 1.1.1.90 | Aryl-alcohol dehydrogenase | MetaCyc: 3-Chlorotoluene degradation II. |
| 1.1.1.96 | Diiodophenylpyruvate reductase | IUBMB comments: Substrates contain an aromatic ring with a pyruvate side chain. The most active substrates are halogenated derivatives. Compounds with hydroxy or amino groups in the 3 or 5 position are inactive. |
| 1.1.2.7 | Methanol dehydrogenase (cytochrome c) | IUBMB comments: Acts on a wide range of primary alcohols, including chloroethanol. MetaCyc: 1,2-Dichloroethane degradation. KEGG: Chloroalkane and chloroalkene degradation. |
| 1.1.2.8 | Alcohol dehydrogenase (cytochrome c) | KEGG: Chloroalkane and chloroalkene degradation. |
| 1.1.5.12 | D-Lactate dehydrogenase (quinone) | MetaCyc: 2-Chloroacrylate degradation I. |
| 1.11.1.10 | **Chloride peroxidase** | IUBMB comments: Brings about the chlorination of a range of organic molecules, forming stable C-Cl bonds. Enzymes of this type are either heme-thiolate proteins, or contain vanadate. A secreted enzyme produced by the ascomycetous fungus *Caldariomyces fumago* (*Leptoxyphium fumago*) is an example of the heme-thiolate type. It catalyses the production of hypochlorous acid by transferring one oxygen atom from H_2_O_2_ to chloride. At a separate site it catalyses the chlorination of activated aliphatic and aromatic substrates, via HClO and derived chlorine species. In the absence of halides, it shows peroxidase (e.g. phenol oxidation) and peroxygenase activities. The latter inserts oxygen from H_2_O_2_ into, for example, styrene (side chain epoxidation) and toluene (benzylic hydroxylation), however, these activities are less pronounced than its activity with halides. Has little activity with non-activated substrates such as aromatic rings, ethers or saturated alkanes. The chlorinating peroxidase produced by *ascomycetous* fungi (e.g. *Curvularia inaequalis*) is an example of a vanadium chloroperoxidase, and is related to bromide peroxidase (EC 1.11.1.18). It contains vanadate and oxidizes chloride, bromide and iodide into hypohalous acids. In the absence of halides, it peroxygenates organic sulfides and oxidizes ABTS [2,2'-azinobis(3-ethylbenzthiazoline-6-sulfonic acid)] but no phenols. |
| 1.11.1.18 | **Bromide peroxidase** | IUBMB comments: Bromoperoxidases of red and brown marine algae (Rhodophyta and Phaeophyta) contain vanadate. Bromination of a range of organic molecules such as sesquiterpenes, forming stable C-Br bonds. Also oxidize iodides. |
| 1.11.1.8 | **Iodide peroxidase** | IUBMB comments: Thyroid peroxidase catalyses both the iodination of tyrosine residues in thyroglobulin (forming mono- and di-iodinated forms) and their coupling to form either L-thyroxine or triiodo-L-thyronine. KEGG: Tyrosine metabolism. MetaCyc: Thyroid hormone biosynthesis. |
| 1.13.11.1 | Catechol 1,2-dioxygenase | BRENDA: 3-Chlorocatechol degradation. KEGG: Chlorocyclohexane, chlorobenzene and fluorobenzoate degradation. |
| 1.13.11.2 | Catechol 2,3-dioxygenase | KEGG: Chlorocyclohexane and chlorobenzene degradation. |
| 1.13.11.36 | Chloridazon-catechol dioxygenase | IUBMB comments: Involved in the breakdown of the herbicide chloridazon. |
| 1.13.11.37 | Hydroxyquinol 1,2-dioxygenase | KEGG: Chlorocyclohexane and chlorobenzene degradation. MetaCyc: 2,4,5-trichlorophenoxyacetate degradation. |
| 1.13.11.39 | Biphenyl-2,3-diol 1,2-dioxygenase | KEGG: Chlorocyclohexane and chlorobenzene degradation. |
| 1.13.11.49 | **Chlorite O2-lyase** | IUBMB comments: Reaction occurs in the reverse direction in chlorate- and perchlorate-reducing bacteria. There is no activity when chlorite is replaced by hydrogen peroxide, perchlorate, chlorate or nitrite. The term 'chlorite dismutase' is misleading as the reaction does not involve dismutation/disproportionation. Contains iron and protoheme IX. MetaCyc: Chlorate reduction, perchlorate reduction. |
| 1.13.11.66 | Hydroquinone 1,2-dioxygenase | KEGG: Chlorocyclohexane and chlorobenzene degradation. |
| 1.13.11.76 | 2-Amino-5-chlorophenol 1,6-dioxygenase | MetaCyc: 4-Chloronitrobenzene degradation. |
| 1.13.11.8 | Protocatechuate 4,5-dioxygenase | MetaCyc: [3,4-Dichlorobenzoate degradation](about:blank), [3-chlorobenzoate degradation II (via protocatechuate)](about:blank). |
| 1.13.12.17 | *Dichloroarcyriaflavin A synthase* | IUBMB comments: The conversion of dichlorochromopyrrolate to dichloroarcyriaflavin A is a complex process that involves two enzyme components. RebP is an NAD-dependent cytochrome P-450 oxygenase that performs an aryl-aryl bond formation yielding the six-ring indolocarbazole scaffold. Along with RebC, a flavin-dependent hydroxylase, it also catalyses the oxidative decarboxylation of both carboxyl groups. The presence of RebC ensures that the only product is the rebeccamycin aglycone dichloroarcyriaflavin A. The enzymes are similar, but not identical, to StaP and StaC, which are involved in the synthesis of staurosporine. MetaCyc: Rebeccamycin biosynthesis. |
| 1.14.11.43 | (*S*)-Dichlorprop dioxygenase (2-oxoglutarate) | IUBMB comments: Fe^2+-^dependent enzyme. The enzymes from the Gram-negative bacteria *Delftia acidovorans* MC1 and *Sphingomonas herbicidovorans* MH are involved in the degradation of the (*S*)-enantiomer of the phenoxyalkanoic acid herbicides mecoprop and dichlorprop. |
| 1.14.11.44 | (*R*)-Dichlorprop dioxygenase (2-oxoglutarate) | IUBMB comments: Fe^2+^ dependent enzyme. The enzymes from the Gram-negative bacteria *Delftia acidovorans* MC1 and *Sphingomonas herbicidovorans* MH are involved in the degradation of the (*R*)-enantiomer of the phenoxyalkanoic acid herbicides mecoprop and dichlorprop. |
| 1.14.12.3 | B[enzene 1,2-dioxygenase](https://www.brenda-enzymes.org/enzyme.php?ecno=1.14.12.3#reactschemes) | KEGG: Chlorocyclohexane and chlorobenzene degradation. |
| 1.14.12.10 | Benzoate 1,2-dioxygenase | KEGG: Fluorobenzoate degradation. |
| 1.14.12.11 | Toluene dioxygenase | IUBMB comments: A system containing a reductase which is an iron-sulfur flavoprotein (FAD), an iron-sulfur oxygenase, and a ferredoxin. Some other aromatic compounds, including ethylbenzene, 4-xylene and some halogenated toluenes, are converted into the corresponding cis-dihydrodiols. KEGG: Chloroalkane and chloroalkene degradation. |
| 1.14.12.13 | **2-Halobenzoate 1,2-dioxygenase** | IUBMB comments: A multicomponent enzyme system composed of a dioxygenase component and an electron transfer component. The latter contains FAD. The enzyme, characterized from the bacterium *Burkholderia cepacia* 2CBS, has a broad substrate specificity. Substrates include 2-fluorobenzoate, 2-chlorobenzoate, 2-bromobenzoate, and 2-iodobenzoate, which are processed in this order of preference. KEGG: Fluorobenzoate degradation. MetaCyc: [2-Chlorobenzoate degradation](about:blank). |
| 1.14.12.23 | Nitroarene dioxygenase | IUBMB comments: It can typically act on many different nitroaromatic compounds, including chlorinated species. Enzymes found in different strains may have different substrate preferences. Requires Fe^2+^. MetaCyc: 1-Chloro-2-nitrobenzene degradation pathway. |
| 1.14.12.26 | Chlorobenzene dioxygenase | IUBMB comments: This bacterial enzyme is a class IIB dioxygenase, comprising three components - a heterodimeric terminal dioxygenase, a ferredoxin protein, and a ferredoxin reductase. The enzyme acts on a range of aromatic compounds, including mono-, di-, tri-, and tetra-chlorinated benzenes and toluenes. KEGG: Chlorocyclohexane and chlorobenzene degradation. MetaCyc: 1,2,4,5-tetrachlorobenzene degradation, 1,2,4-trichlorobenzene degradation, 1,2-dichlorobenzene degradation, 1,3-dichlorobenzene degradation, 1,4-dichlorobenzene degradation, 2,4-dichlorotoluene degradation, 2,5-dichlorotoluene degradation, 3,4-dichlorotoluene degradation, 3-chlorotoluene degradation I, chlorobenzene degradation. |
| 1.14.12.18 | [Biphenyl 2,3-dioxygenase](https://www.brenda-enzymes.org/enzyme.php?ecno=1.14.12.18#reactschemes) | KEGG: Dioxin degradation (4-chlorobiphenyl and PCB degradation). |
| 1.14.12.9 | **4-Chlorophenylacetate 3,4-dioxygenase** | IUBMB comments: A system, containing a reductase and an iron-sulfur oxygenase, and no independent ferredoxin. Requires Fe^2+^. Also acts on 4-bromophenyl acetate. |
| 1.14.13.1 | Salicylate 1-monooxygenase | KEGG: Dioxin degradation (PCDF degradation). MetaCyc: Chlorosalicylate degradation pathway. |
| 1.14.13.2 | *4-Hydroxybenzoate 3-monooxygenase* | MetaCyc: 4-Chlorobenzoate degradation. Polybrominated dihydroxylated diphenyl ethers biosynthesis. |
| 1.14.13.20 | 2,4-Dichlorophenol 6-monooxygenase | IUBMB comments: A flavoprotein (FAD). Also acts on 4-chlorophenol and 4-chloro-2-methylphenol. KEGG: Chlorocyclohexane and chlorobenzene degradation. MetaCyc: 2,4-Dichlorophenoxyacetate degradation, 4-chloro-2-methylphenoxyacetate degradation. |
| 1.14.13.24 | 3-Hydroxybenzoate 6-monooxygenase | MetaCyc: 3-Chlorobenzoate degradation III (via gentisate). |
| 1.14.13.43 | *Questin monooxygenase* | IUBMB comments: The enzyme cleaves the anthraquinone ring of questin to form a benzophenone. Involved in the biosynthesis of the seco-anthraquinone (+)-geodin. MetaCyc: Geodin biosynthesis pathway. UniProtKB: Questin oxidase; part of the gene cluster that mediates the biosynthesis of geodin, an intermediate in the biosynthesis of other natural products |
| 1.14.13.50 | **Pentachlorophenol 4-monooxygenase** | IUBMB comments: A flavoprotein (FAD). The enzyme displaces a diverse range of substituents from the 4-position of polyhalogenated phenols but requires that a halogen substituent be present at the 2-position. If C-4 carries a halogen substituent, reaction 1 is catalysed; if C-4 is unsubstituted, reaction 2 is catalysed. KEGG: Chlorocyclohexane and chlorobenzene degradation, fluorobenzoate degradation. MetaCyc: Pentachlorophenol degradation. |
| 1.14.13.69 | Alkene monooxygenase | KEGG: Chloroalkane and chloroalkene degradation. MetaCyc: Chloroethene degradation. |
| 1.14.13.7 | Phenol 2-monooxygenase (NADPH) | IUBMB comments: A flavoprotein (FAD). The enzyme from the fungus *Trichosporon cutaneum* has a broad substrate specificity, and has been reported to catalyse the hydroxylation of a variety of substituted phenols, such as fluoro-, chloro-, amino- and methyl-phenols and also dihydroxybenzenes. cf. EC 1.14.14.20, phenol 2-monooxygenase (FADH_2_). MetaCyc: Chlorinated phenols degradation. |
| 1.14.13.244 | P[henol 2-monooxygenase (NADH)](https://www.brenda-enzymes.org/enzyme.php?ecno=1.14.13.244#reactschemes) | KEGG: Chlorocyclohexane and chlorobenzene degradation. |
| 1.14.14.1 | Unspecific monooxygenase | IUBMB comments: A group of P-450 heme-thiolate proteins, acting on a wide range of substrates including many xenobiotics, steroids, fatty acids, vitamins and prostaglandins. KEGG: Metabolism of xenobiotics by cytochrome P450 (trichloroethylene metabolism). |
| 1.14.14.15 | *(3S)-3-Amino-3-(3-chloro-4-hydroxyphenyl)propanoyl-[peptidyl-carrier protein SgcC2] monooxygenase* | IUBMB comments: The enzyme from the bacterium *Streptomyces globisporus* is involved in the biosynthesis of the (*S*)-3-chloro-5-hydroxy-beta-tyrosine moiety prior to incorporation into the chromoprotein antitumor antibiotic C-1027. |
| 1.14.14.172 | **3,5,6-Trichloropyridin-2-ol monooxygenase** | IUBMB comments: The enzyme, characterized from a number of bacterial species, participates in the degradation of 3,5,6-trichloropyridin-2-ol (TCP), a metabolite of the common organophosphorus insecticide chlorpyrifos. The enzyme is a multifunctional flavin-dependent monooxygenase that displaces three chlorine atoms by attacking three different positions in the substrate. Each reaction catalysed by the enzyme displaces a single chlorine and results in formation of a dione, which must be reduced by FADH_2_ before the monooxygenase could catalyse the next step. The large amount of FADH_2_ that is required is generated by a dedicated flavin reductase (TcpX). cf. EC 1.14.14.173, 2,4,6-trichlorophenol monooxygenase. MetaCyc: 3,5,6-Trichloro-2-pyridinol degradation. |
| 1.14.14.173 | **2,4,6-Trichlorophenol monooxygenase** | IUBMB comments: The enzyme, characterized from a number of bacterial species, participates in the degradation of 2,4,6-trichlorophenol, a compound that has been used for decades as a wood preservative. The enzyme is a multifunctional flavin-dependent monooxygenase that displaces two chlorine atoms by attacking two different positions in the substrate. Each reaction catalysed by the enzyme displaces a single chlorine and results in formation of a dione, which must be reduced by FADH_2_ before the monooxygenase could catalyse the second reaction. The large amount of FADH_2_ that is required is generated by a dedicated flavin reductase (TcpB). cf. EC 1.14.14.172, 3,5,6-trichloropyridin-2-ol monooxygenase. KEGG: [Chlorocyclohexane and chlorobenzene degradation](about:blank). MetaCyc: 2,4,6-Trichlorophenol degradation. |
| 1.14.15.23 | Chloroacetanilide N-alkylformylase | IUBMB comments: The enzyme, characterized from the bacterium *Sphingomonas sp.* DC-6, initiates the degradation of several chloroacetanilide herbicides, including alachlor, acetochlor, and butachlor. The enzyme is a Rieske non-heme iron oxygenase, and requires a ferredoxin and EC 1.18.1.3, ferredoxin---NAD+ reductase, for activity. |
| 1.14.15.26 | Toluene methyl-monooxygenase | IUBMB comments: The enzyme, characterized from several *Pseudomonas* strains, catalyses the first step in the degradation of toluenes and xylenes. It has a broad substrate specificity and is also active with substituted compounds, such as chlorotoluenes. MetaCyc: 3-chlorotoluene degradation II. |
| 1.14.19.49 | ***Tetracycline 7-halogenase*** | IUMBM comments: The enzyme, characterized from the bacterium *Streptomyces aureofaciens,* is a member of the flavin-dependent halogenase family. The enzyme forms a lysine chloramine intermediate on an internal lysine residue before transferring the chlorine to the substrate. It is stereo-selective for the 4S (natural) isomer of tetracycline. FADH_2_ is provided by a dedicated EC 1.5.1.36, flavin reductase (NADH). MetaCyc: Chlorotetracycline biosynthesis. KEGG: Tetracycline biosynthesis. |
| 1.14.19.55 | ***4-Hydroxybenzoate brominase (decarboxylating)*** | IUBMB comments: Contains FAD. The enzyme, described from epiphytic marine bacteria of the genera *Pseudoalteromonas* and *Marinomonas*, is an unusual single-component FAD-dependent halogenase that contains a distinct NAD(P)H binding domain and does not require an additional flavin reductase for activity. The enzyme catalyses a bromination of its substrate, followed by a second bromination concurrent with decarboxylation. MetaCyc: Polybrominated phenols biosynthesis. |
| 1.14.19.56 | ***1H-pyrrole-2-carbonyl-[peptidyl-carrier protein] chlorinase*** | IUBMB comments: The enzyme, characterized from the bacterium *Pseudomonas protegens* Pf-5, is a flavin-dependent chlorinase that participates in the biosynthesis of the antibacterial and antifungal compound pyoluteorin. MetaCyc: Pyrrolomycin biosynthesis. |
| 1.14.19.57 | ***1H-pyrrole-2-carbonyl-[peptidyl-carrier protein] brominase*** | IUBMB comments: The enzyme, characterized from marine bacteria of the *Pseudoalteromonas* genus, belongs to a family of FAD-dependent halogenases that act on acyl-carrier protein-tethered substrates. It catalyses three successive rounds of bromination. While the order has not been verified, it is believed to resemble that of EC 1.14.19.56, S-(1H-pyrrole-2-carbonyl)-[peptidyl-carrier protein] chlorinase, due to significant sequence homology. Reduced FAD is provided in situ by a dedicated reductase and diffuses into the active site, where it reacts with the oxygen and bromide ion, resulting in formation of a bromoamine intermediate on a catalytic lysine side chain, and the eventual transfer of the bromide to the substrate. The enzyme from *Pseudoalteromonas luteoviolacea* 2ta16 is specific for bromide and does not accept chloride. |
| 1.14.19.58 | ***Tryptophan 5-halogenase*** | IUBMB comments: A flavin-dependent halogenase. The enzyme from the bacterium *Streptomyces rugosporus* catalyses halogenation of the C-5 position of tryptophan during the biosynthesis of the antibiotic compound pyrroindomycin B. It utilizes molecular oxygen to oxidize the FADH_2_ cofactor, giving C4a-hydroperoxyflavin, which then reacts with chloride to produce a hypochlorite ion. The latter reacts with an active site lysine to generate a chloramine, which chlorinates the substrate. cf. EC 1.14.19.59, tryptophan 6-halogenase and EC 1.14.19.9, tryptophan 7-halogenase. |
| 1.14.19.59 | ***Tryptophan 6-halogenase*** | IUBMB comments: The enzyme is a flavin-dependent halogenase that has been described from several bacterial species. It utilizes molecular oxygen to oxidize the FADH_2_ cofactor, giving C4a-hydroperoxyflavin, which then reacts with chloride to produce a hypochlorite ion. The latter reacts with an active site lysine to generate a chloramine, which chlorinates the substrate. cf. EC 1.14.19.58, tryptophan 5-halogenase, and EC 1.14.19.9, tryptophan 7-halogenase. |
| 1.14.19.60 | ***7-Chloro-L-tryptophan 6-halogenase*** | IUBMB comments: An FAD-dependent halogenase. The enzyme, characterized from the bacterium *Kutzneria* sp. 744, works in tandem with EC 1.14.19.9, tryptophan 7-halogenase, (ktzQ) to generate 6,7-dichloro-L-tryptophan, which is incorporated as a pyrroloindoline in the kutznerides family of natural products. It has a 120-fold preference for 7-chloro-L-tryptophan over L-tryptophan as substrate. |
| 1.14.19.9 | ***Tryptophan 7-halogenase*** | IUBMB comments: A flavin-dependent halogenase. The enzyme from the bacterium *Lechevalieria aerocolonigenes* catalyses the initial step in the biosynthesis of rebeccamycin. It utilizes molecular oxygen to oxidize the FADH_2_ cofactor, giving C4a-hydroperoxyflavin, which then reacts with chloride to produce a hypochlorite ion. The latter reacts with an active site lysine to generate a chloramine, which chlorinates the substrate. Also acts on bromide ion. cf. EC 1.14.19.58, tryptophan 5-halogenase, and EC 1.14.19.59, tryptophan 6-halogenase. MetaCyc: Pyrrolnitrin biosynthesis, rebeccamycin biosynthesis. |
| 1.14.20.14 | ***Hapalindole-type alkaloid chlorinase*** | IUBMB comments: The enzyme, characterized from hapalindole-type alkaloids-producing cyanobacteria, is a specialized iron(II)/2-oxoglutarate-dependent oxygenase that catalyses the chlorination of its substrates in a reaction that requires oxygen, chloride ions, iron(II) and 2-oxoglutarate. |
| 1.14.20.15 | ***L-Threonyl-[L-threonyl-carrier protein] 4-chlorinase*** | IUBMB comments: The enzyme, characterized from the bacterium *Pseudomonas syringae*, participates in syringomycin E biosynthesis. The enzyme is a specialized iron(II)/2-oxoglutarate-dependent oxygenase that catalyses the chlorination of its substrate in a reaction that requires oxygen, chloride ions, ferrous iron and 2-oxoglutarate. |
| 1.14.99.65 | [*4-Amino-L-phenylalanyl-[CmlP-peptidyl-carrier-protein] 3-hydroxylase*](https://www.brenda-enzymes.org/enzyme.php?ecno=1.14.99.65#reactschemes) | IUBMB comments: The enzyme, characterized from the bacterium *Streptomyces venezuelae*, participates in the biosynthesis of the antibiotic chloramphenicol. MetaCyc: [Chloramphenicol biosynthesis](about:blank). |
| 1.14.99.67 | [*Alpha-N-dichloroacetyl-p-aminophenylserinol N-oxygenase*](https://www.brenda-enzymes.org/enzyme.php?ecno=1.14.99.67#reactschemes) | IUBMB comments: The enzyme, isolated from the bacterium *Streptomyces venezuelae*, is involved in the biosynthesis of the antibiotic chloramphenicol. |
| 1.17.1.10 | Formate dehydrogenase (NADP+) | MetaCyc: [Carbon tetrachloride degradation I](https://biocyc.org/META/NEW-IMAGE?type=PATHWAY&object=PWY-5372)I. |
| 1.18.1.3 | F[erredoxin-NAD+ reductase](https://www.brenda-enzymes.org/enzyme.php?ecno=1.18.1.3#reactschemes) | IUBMB comments: See EC 1.14.15.23. UniProtKB: Chloroacetanilide N-alkylformylase, ferredoxin reductase component, EC 1.18.1.3. Component of the chloroacetanilide N-alkylformylase multicomponent enzyme system involved in the degradation of chloroacetanilide herbicides (N-alkoxyalkyl-N-chloroacetyl-substituted aniline derivatives) |
| 1.18.6.1 | Nitrogenase | KEGG: Chloroalkane and chloroalkene degradation. |
| 1.2.1.28 | Benzaldehyde dehydrogenase (NAD+) | MetaCyc: 3-Chlorotoluene degradation II. |
| 1.2.1.3 | Aldehyde dehydrogenase (NAD+) | KEGG: Chloroalkane and chloroalkene degradation. |
| 1.2.1.32 | Aminomuconate-semialdehyde dehydrogenase | MetaCyc: 4-Chloronitrobenzene degradation. |
| 1.2.1.4 | Aldehyde dehydrogenase (NADP+) | MetaCyc: 1,2-Dichloroethane degradation. |
| 1.2.1.46 | Formaldehyde dehydrogenase | KEGG: Chloroalkane and chloroalkene degradation. |
| 1.2.1.5 | Aldehyde dehydrogenase [NAD(P)+] | KEGG: Metabolism of xenobiotics by cytochrome P450 (trichloroethylene degradation). |
| 1.2.1.61 | 4-Hydroxymuconic-semialdehyde dehydrogenase | KEGG: Chlorocyclohexane and chlorobenzene degradation. |
| 1.2.1.69 | *Fluoroacetaldehyde dehydrogenase* | MetaCyc: Fluoroacetate and fluorothreonine biosynthesis. |
| 1.2.7.4 | Anaerobic carbon-monoxide dehydrogenase | MetaCyc: Carbon tetrachloride degradation II. |
| 1.2.98.1 | Formaldehyde dismutase | KEGG: Chloroalkane and chloroalkene degradation. |
| 1.21.1.1 | **Iodotyrosine deiodinase** | IUBMB comments: The enzyme activity has only been demonstrated in the direction of 3-deiodination. The enzyme activity has only been demonstrated in the direction of 3-deiodination. Requires FMN. |
| 1.21.1.2 | **2,4-Dichlorobenzoyl-CoA reductase** | IUBMB comments: The enzyme, characterized from *Corynebacterium* strains able to grow on 2,4-dichlorobenzoate, forms part of the 2,4-dichlorobenzoate degradation pathway. KEGG: Fluorobenzoate degradation. |
| 1.21.4.5 | **Tetrachlorohydroquinone reductive dehalogenase** | IUBMB comments: The enzyme, characterized from the bacterium *Sphingobium chlorophenolicum*, converts tetrachlorohydroquinone to 2,6-dichlorohydroquinone. KEGG: Chlorocyclohexane and chlorobenzene degradation. |
| 1.21.98.2 | *Dichlorochromopyrrolate synthase* | IUBMB comments: This enzyme catalyses a step in the biosynthesis of rebeccamycin, an indolocarbazole alkaloid produced by the bacterium *Lechevalieria aerocolonigenes*. The enzyme is a dimeric heme-protein oxidase that catalyses the oxidative dimerization of two L-tryptophan-derived molecules to form dichlorochromopyrrolic acid, the precursor for the fused six-ring indolocarbazole scaffold of rebeccamycin. Contains one molecule of heme b per monomer, as well as non-heme iron that is not part of an iron-sulfur center. In vivo the enzyme uses hydrogen peroxide, formed by the enzyme upstream in the biosynthetic pathway (EC 1.4.3.23, 7-chloro-L-tryptophan oxidase) as the electron acceptor. However, the enzyme is also able to catalyse the reaction using molecular oxygen. MetaCyc: Rebeccamycin biosynthesis. |
| 1.21.99.3 | **Thyroxine 5-deiodinase** | IUBMB comments: The enzyme activity has only been demonstrated in the direction of 5-deiodination. This removal of the 5-iodine, i.e. from the inner ring, largely inactivates the hormone thyroxine. |
| 1.21.99.4 | **Thyroxine 5'-deiodinase** | IUBMB comments: The enzyme activity has only been demonstrated in the direction of 5'-deiodination, which renders the thyroid hormone more active. The enzyme consists of type I and type II enzymes, both containing selenocysteine, but with different kinetics. For the type I enzyme the first reaction is a reductive deiodination converting the -Se-H group of the enzyme into an -Se-I group; the reductant then reconverts this into -Se-H, releasing iodide. |
| 1.21.99.5 | **Tetrachloroethene reductive dehalogenase** | IUBMB comments: This enzyme allows the common pollutant tetrachloroethene to support bacterial growth and is responsible for disposal of a number of chlorinated hydrocarbons. The reaction occurs in the reverse direction. The enzyme also reduces trichloroethene to dichloroethene. Although the physiological reductant is unknown, the supply of reductant in some organisms involves menaquinol, which is reduced by molecular hydrogen via the action of EC 1.12.5.1, hydrogen:quinone oxidoreductase. The enzyme contains a corrinoid and two iron-sulfur clusters. Methylviologen can act as electron donor in vitro. KEGG: Chloroalkane and chloroalkene degradation. MetaCyc: Tetrachloroethene degradation. |
| 1.3.1.103 | 2-Haloacrylate reductase | IUBMB comments: The enzyme acts in the degradation pathway of unsaturated organohalogen compounds by the bacterium *Burkholderia sp*. WS. MetaCyc: 2-Chloroacrylate degradation I. |
| 1.3.1.119 | Chlorobenzene dihydrodiol dehydrogenase | IUBMB comments: This bacterial enzyme can transform various dihydrodiols of chlorobenzenes into the respective catechols, including the dihydrodiols of mono-, di-, tri-, and tetra-chlorinated benzenes. It also accepts the dihydrodiols of various chlorotoluenes. Substrates for the enzyme are generated by the broad spectrum EC 1.14.12.26, chlorobenzene dioxygenase. KEGG: Chlorocyclohexane and chlorobenzene degradation. MetaCyc: Chlorocyclohexane and chlorobenzene degradation. 1,2,4-trichlorobenzene degradation, 1,2-dichlorobenzene degradation, 1,3-dichlorobenzene degradation, 1,4-dichlorobenzene degradation, 2,4-dichlorotoluene degradation, 2,5-dichlorotoluene degradation, 3,4-dichlorotoluene degradation, 3-chlorotoluene degradation I, chlorobenzene degradation. |
| 1.3.1.19 | Cis-1,2-dihydrobenzene-1,2-diol dehydrogenase | KEGG: Chlorocyclohexane and chlorobenzene degradation. |
| 1.3.1.25 | 1,6-Dihydroxycyclohexa-2,4-diene-1-carboxylate dehydrogenase | KEGG: Fluorobenzoate degradation. |
| 1.3.1.32 | Maleylacetate reductase | KEGG: Chlorocyclohexane and chlorobenzene degradation, Fluorobenzoate degradation. MetaCyc: 3-Chlorocatechol degradation. 1,4-dichlorobenzene degradation, 2,4,5-trichlorophenoxyacetate degradation, 2,4,6-trichlorophenol degradation, 3,4,6-trichlorocatechol degradation, 3,5-dichlorocatechol degradation, 3-chlorocatechol degradation I (ortho), 3-chlorocatechol degradation II (ortho), 4,5-dichlorocatechol degradation, 4-chlorocatechol degradation, chlorosalicylate degradation, gamma-hexachlorocyclohexane degradation, pentachlorophenol degradation. |
| 1.3.1.56 | [Cis-2,3-dihydrobiphenyl-2,3-diol dehydrogenase](https://www.brenda-enzymes.info/enzyme.php?ecno=1.3.1.56#reactschemes) | KEGG: Dioxin degradation (4-chlorobiphenyl and PCB degradation). |
| 1.3.1.121 | [*4-Amino-4-deoxyprephenate dehydrogenase*](https://www.brenda-enzymes.org/enzyme.php?ecno=1.3.1.121#reactschemes) | IUBMB comments: The enzyme, characterized from the bacteria *Streptomyces venezuelae* and *Streptomyces pristinaespiralis*, participates in the biosynthesis of the antibiotics chloramphenicol and pristinamycin IA, respectively. cf. EC 1.3.1.12, prephenate dehydrogenase. MetaCyc: [Chloramphenicol biosynthesis](about:blank). |
| 1.3.8.14 | *L-Prolyl-[peptidyl-carrier protein] dehydrogenase* | MetaCyc: Brominated pyrroles biosynthesis, pyoluteorin biosynthesis. |
| 1.4.3.23 | *7-Chloro-L-tryptophan oxidase* | IUBMB comments: This enzyme catalyses a step in the biosynthesis of rebeccamycin, an indolocarbazole alkaloid produced by the bacterium *Lechevalieria aerocolonigenes*. During catalysis, the bound FAD is reoxidized at the expense of molecular oxygen, producing one molecule of hydrogen peroxide. The enzyme shows significant preference for 7-chloro-L-tryptophan over L-tryptophan. MetaCyc: Rebeccamycin biosynthesis. |
| 1.6.5.7 | 2-Hydroxy-1,4-benzoquinone reductase | IUBMB comments: A flavoprotein (FMN) that differs in substrate specificity from other quinone reductases. The enzyme in *Burkholderia cepacia* is inducible by 2,4,5-trichlorophenoxyacetate. KEGG: Chlorocyclohexane and chlorobenzene degradation. MetaCyc: 2,4,5-Trichlorophenoxyacetate degradation. |
| 1.7.1.16 | Nitrobenzene nitroreductase | IUBMB comments: Contains FMN. The enzyme, characterized from *Pseudomonas* species, catalyses two successive reductions of nitrobenzene, via a nitrosobenzene intermediate. It is also active on 1-chloro-4-nitrobenzene.  UniProtKB: Chloronitrobenzene nitroreductase, EC 1.7.1.16. Involved in the biodegradation of chlorinated nitroaromatic compounds. Catalyzes the reduction of 4-chloronitrobenzene to yield 1-hydroxylamino-4-chlorobenzene. |
| 1.8.1.5 | 2-Oxopropyl-CoM reductase (carboxylating) | MetaCyc: Ethene and chloroethene degradation. |
| 1.8.5.7 | Glutathionyl-hydroquinone reductase | IUBMB comments: This type of enzymes, which are found in bacteria, halobacteria, fungi, and plants, catalyse the glutathione-dependent reduction of glutathionyl-hydroquinones. The enzyme from the bacterium *Sphingobium chlorophenolicum* can act on halogenated substrates such as 2,6-dichloro-3-(glutathione-S-yl)-hydroquinone and 2,3,5-trichloro-6-(glutathione-S-yl)-hydroquinone. Substrates for these enzymes are often formed spontaneously by interaction of benzoquinones with glutathione. MetaCyc: Pentachlorophenol degradation. |
| 1.97.1.1 | Chlorate reductase | MetaCyc: Chlorate reduction. |
| 1.97.1.10 | Thyroxine 5'-deiodinase | Transferred to EC 1.21.99.4 |
| 1.97.1.8 | Tetrachloroethene reductive dehalogenase | Transferred to EC 1.21.99.5 |
| 2.1.1.136 | Chlorophenol O-methyltransferase | IUBMB comments: The enzyme from the fungus *Trichoderma sp. virgatum*, when cultured in the presence of halogenated phenol, also acts on a range of mono-, di- and trichlorophenols. |
| 2.1.1.164 | *Demethylrebeccamycin-D-glucose O-methyltransferase* | IUBMB comments: Catalyses the last step in the biosynthesis of rebeccamycin, an indolocarbazole alkaloid produced by the bacterium *Lechevalieria aerocolonigenes*. The enzyme is able to use a wide variety substrate, tolerating variation on the imide heterocycle, deoxygenation of the sugar moiety, and even indolocarbazole glycoside anomers. The enzyme is a member of the general acid/base-dependent O-methyltransferase family. MetaCyc: Rebeccamycin biosynthesis. |
| 2.1.1.165 | ***Methyl halide transferase*** | IUBMB comments: This enzyme contributes to the methyl halide emissions from *Arabidopsis.* MetaCyc: Methylhalides biosynthesis (plants). |
| 2.1.1.26 | Iodophenol O-methyltransferase | No comments. Reaction scheme: [malonyl-CoA](https://www.brenda-enzymes.org/ligand.php?brenda_ligand_id=76) + [3,4-dichloroaniline](https://www.brenda-enzymes.org/ligand.php?brenda_ligand_id=4204) = [CoA](https://www.brenda-enzymes.org/ligand.php?brenda_ligand_id=18) + [N-(3,4-dichlorophenyl)-malonamate](https://www.brenda-enzymes.org/ligand.php?brenda_ligand_id=45682) |
| 2.1.1.283 | *Emodin O-methyltransferase* | IUBMB comments: The enzyme is involved in biosynthesis of the seco-anthraquinone (+)-geodin. MetaCyc: Geodin biosynthesis. UniProtKB: Part of the gene cluster that mediates the biosynthesis of geodin, an intermediate in the biosynthesis of other natural products. |
| 2.2.1.8 | *Fluorothreonine transaldolase* | IUBMB comments: A pyridoxal phosphate protein. Can also convert chloroacetaldehyde into4-chloro-L-threonine. MetaCyc: Fluoroacetate and fluorothreonine biosynthesis. |
| 2.3.1.114 | 3,4-Dichloroaniline N-malonyltransferase | No comments. Reaction scheme: Malonyl-CoA + 3,4-dichloroaniline = CoA + N-(3,4-dichlorophenyl)-malonamate |
| 2.3.1.28 | Chloramphenicol O-acetyltransferase | UniProtKB: This enzyme is an effector of chloramphenicol resistance in bacteria. Conversion of chloramphenicol to chloramphenicol 3-acetate. |
| 2.4.1.17 | Glucuronosyltransferase | KEGG: Metabolism of xenobiotics by cytochrome P450 (trichloroethylene and trichloroethanol-glucuronide metabolism). |
| 2.4.1.311 | *Chloroorienticin B synthase* | IUBMB comments: The enzyme from the bacterium *Amycolatopsis orientalis* is Involved in the biosynthesis of the glycopeptide antibiotic chloroeremomycin. MetaCyc: Biosynthesis of vancomycin group antibiotics. |
| 2.4.2.1 | *Purine-nucleoside phosphorylase* | MetaCyc: Fluoroacetate and fluorothreonine biosynthesis. [Salinosporamide A biosynthesis](about:blank). |
| 2.5.1.18 | Glutathione transferase | IUBMB comments: A group of enzymes of broad specificity. R may be an aliphatic, aromatic or heterocyclic group; X may be a sulfate, nitrile or halide group. KEGG: Metabolism of xenobiotics by cytochrome P450 (trichloroethylene, 1,1-dichloroethylene, bromobenzene, and 1,2-dibromoethane degradation). MetaCyc: Pentachlorophenol degradation. |
| 2.5.1.63 | ***Adenosyl-fluoride synthase*** | MetaCyc: Fluoroacetate and fluorothreonine biosynthesis. |
| 2.5.1.94 | ***Adenosyl-chloride synthase*** | IUBMB comments: This enzyme, isolated from the marine bacterium *Salinispora tropica*, catalyses an early step in the pathway leading to biosynthesis of the proteosome inhibitor salinosporamide A. The enzyme is very similar to EC 2.5.1.63, adenosyl-fluoride synthase, but does not accept fluoride. MetaCyc: Salinosporamide A biosynthesis. |
| 2.6.1.24 | Diiodotyrosine transaminase | IUBMB comments: A pyridoxal-phosphate protein. Also acts on 3,5-dichloro-, 3,5-dibromo- and 3-iodo-L-tyrosine, thyroxine and triiodothyronine. |
| 2.6.1.85 | [*Aminodeoxychorismate synthase*](https://www.brenda-enzymes.org/enzyme.php?ecno=2.6.1.85#reactschemes) | MetaCyc: [Chloramphenicol biosynthesis](about:blank). |
| 3.1.1.45 | Carboxymethylenebutenolidase | KEGG: Chlorocyclohexane and chlorobenzene degradation, Fluorobenzoate degradation. MetaCyc: 1,4-Dichlorobenzene degradation, 3,4,6-trichlorocatechol degradation, 3,5-dichlorocatechol degradation, 3-chlorocatechol degradation I (ortho), 3-chlorocatechol degradation II (ortho), 4,5-dichlorocatechol degradation, 4-chlorocatechol degradation. |
| 3.1.1.88 | Pyrethroid hydrolase | IUBMB comments: The enzyme is involved in degradation of pyrethroid pesticides. The enzymes from *Sphingobium* sp., *Klebsiella* sp. and *Aspergillus niger* hydrolyse cis-permethrin at approximately equal rate to trans-permethrin. The enzyme from mouse hydrolyses trans-permethrin at a rate about 22-fold higher than cis-permethrin. |
| 3.1.2.23 | 4-Hydroxybenzoyl-CoA thioesterase | IUBMB comments: This enzyme is part of the bacterial 2,4-dichlorobenzoate degradation pathway. MetaCyc: 4-Chlorobenzoate degradation. |
| 3.1.2.29 | Fluoroacetyl-CoA thioesterase | IUBMB comments: Fluoroacetate is extremely toxic. It reacts with CoA to form fluoroacetyl-CoA, which substitutes for acetyl-CoA and reacts with EC 2.3.3.1 (citrate synthase) to produce fluorocitrate, a metabolite of which binds very tightly to EC 4.2.1.3 (aconitase) and halts the TCA cycle. This enzyme hydrolyses fluoroacetyl-CoA before it can react with citrate synthase, and thus confers fluoroacetate resistance on the organisms that produce it. It has been described in the poisonous plant *Dichapetalum cymosum* and the bacterium *Streptomyces cattleya*, both of which are fluoroacetate producers. |
| 3.1.8.1 | Aryldialkylphosphatase | MetaCyc: Chlorpyrifos degradation. |
| 3.1.8.2 | **Diisopropyl-fluorophosphatase** | IUBMB comments: Acts on phosphorus anhydride bonds (such as phosphorus-halide and phosphorus-cyanide) in organophosphorus compounds (including 'nerve gases'). Related to EC 3.1.8.1 aryldialkylphosphatase. |
| 3.3.2.10 | Soluble epoxide hydrolase | KEGG: Chloroalkane and chloroalkene degradation. |
| 3.3.2.9 | Microsomal epoxide hydrolase | KEGG: Metabolism of xenobiotics by cytochrome P450 (bromobenzene metabolism). |
| 3.5.1.131 | 1-Carboxybiuret hydrolase | IUBMB comments: The enzyme, characterized from the bacterium *Pseudomonas* sp. ADP, participates in the degradation of s-triazide herbicides such as atrazine [2-chloro-4-(ethylamino)-6-(isopropylamino)-1,3,5-triazine]. KEGG: Atrazine degradation. |
| 3.5.1.54 | Allophanate hydrolase | IUBMB comments: Along with EC 3.5.2.15 (cyanuric acid amidohydrolase) and EC 3.5.1.84 (biuret amidohydrolase), this enzyme forms part of the cyanuric-acid metabolism pathway, which degrades s-triazide herbicides, such as atrazine [2-chloro-4-(ethylamino)-6-(isopropylamino)-1,3,5-triazine], in bacteria. The yeast enzyme (but not that from green algae) also catalyses the reaction of EC 6.3.4.6, urea carboxylase, thus bringing about the hydrolysis of urea to CO_2_ and NH3 in the presence of ATP and bicarbonate. The enzyme from *Pseudomonas sp.* strain ADP has a narrow substrate specificity, being unable to use the structurally analogous compounds urea, hydroxyurea or methylcarbamate as substrate. KEGG: Atrazine degradation. |
| 3.5.1.84 | Biuret amidohydrolase | IUBMB comments: The enzyme, characterized from the bacterium *Rhizobium leguminosarum* bv. viciae 3841, participates in the degradation of cyanuric acid, an intermediate in the degradation of s-triazide herbicides such as atrazine [2-chloro-4-(ethylamino)-6-(isopropylamino)-1,3,5-triazine]. KEGG: Atrazine degradation. |
| 3.5.2.15 | Cyanuric acid amidohydrolase | IUBMB comments: The enzyme catalyses the ring cleavage of cyanuric acid, an intermediate in the degradation of s-triazide herbicides such as atrazine [2-chloro-4-(ethylamino)-6-(isopropylamino)-1,3,5-triazine]. The enzyme is highly specific for cyanuric acid. KEGG: Atrazine degradation. |
| 3.5.4.42 | N-Isopropylammelide isopropylaminohydrolase | IUBMB comments: Requires Zn^2+^. This bacterial enzyme is involved in degradation of the herbicide atrazine. It can hydrolyse other N-substituted amino dihydroxy-s-triazine molecules, and prefers substrates with linear N-alkyl groups to those with branched alkyl groups. KEGG: Atrazine degradation. MetaCyc: Atrazine degradation I (aerobic). |
| 3.5.4.43 | Hydroxydechloroatrazine ethylaminohydrolase | IUBMB comments: Contains Zn^2+.^ This bacterial enzyme is involved in degradation of the herbicide atrazine. The enzyme has a broad substrate range, and requires a monohydroxylated s-triazine ring with a minimum of one primary or secondary amine substituent and either a chloride or amine leaving group. It catalyses both deamination and dechlorination reactions. KEGG: Atrazine degradation. MetaCyc: Atrazine degradation I (aerobic). |
| 3.5.4.45 | Melamine deaminase | IUBMB comments: The enzyme, isolated from the bacterium *Acidovorax citrulli*, performs the deamination of melamine 15-fold faster than the deamination of ammeline. It also has activity with 2-chloro-4,6-diamino-s-triazine, but has no activity toward halo-substituted triazine ring compounds such as atrazine (cf. EC 3.8.1.8, atrazine chlorohydrolase). KEGG: Atrazine degradation. |
| 3.5.5.6 | Bromoxynil nitrilase | IUBMB comments: Involved in the bacterial degradation of the herbicide bromoxynil. Highly specific. KEGG: Fluorobenzoate degradation. |
| 3.5.99.11 | 2-Aminomuconate deaminase (2-hydroxymuconate-forming) | IUBMB comments: The enzyme, characterized from the bacterium *Comamonas testosteroni* CNB-1, converts 2-aminomuconate to 2-hydroxyhexa-2,4-dienedioate, unlike the enzymes from *Pseudomonas*, which produce (3E)-2-oxohex-3-enedioate (see EC 3.5.99.5, 2-aminomuconate deaminase). The enzyme also acts on 2-amino-5-chloromuconate. MetaCyc: 4-Chloronitrobenzene degradation. |
| 3.5.99.5 | 2-Aminomuconate deaminase | IUBMB comments: 2-Aminomuconate is an intermediate in the bacterial biodegradation of nitrobenzene. The enzyme has been isolated from several species, including *Pseudomonas pseudocaligenes* JS45, *Pseudomonas fluorescens* KU-7, *Pseudomonas sp*. AP3 and *Burkholderia cenocepacia* J2315. The reaction is spontaneous in acid conditions. UniProtKB: 2-Amino-5-chloromuconic acid deaminase EC 3.5.99.5. This protein is involved in the pathway 4-chloronitrobenzene degradation, which is part of Xenobiotic degradation. |
| 3.7.1.8 | [2,6-Dioxo-6-phenylhexa-3-enoate hydrolase](https://www.brenda-enzymes.org/enzyme.php?ecno=3.7.1.8#reactschemes) | KEGG: Dioxin degradation (4-chlorobiphenyl and PCB degradation) |
| 3.8.1.1 | Alkylhalidase | Transferred to EC 3.8.1.5 |
| 3.8.1.10 | **2-Haloacid dehalogenase (configuration-inverting)** | IUBMB comments: Dehalogenates both (*S*)- and (*R*)-2-haloalkanoic acids to the corresponding (*R*)- and (S)-hydroxyalkanoic acids, respectively, with inversion of configuration at C-2. The enzyme from *Pseudomonas sp*. 113 acts on 2-haloalkanoic acids whose carbon chain lengths are five or less. [See also EC 3.8.1.2 (*S*)-2-haloacid dehalogenase, EC 3.8.1.9 (*R*)-2-haloacid dehalogenase and EC 3.8.1.11 2-haloacid dehalogenase (configuration-retaining)]. |
| 3.8.1.11 | **2-Haloacid dehalogenase (configuration-retaining)** | IUBMB comments: Dehalogenates both (*S*)- and (*R*)-2-haloalkanoic acids to the corresponding (*S*)- and (*R*)-hydroxyalkanoic acids, respectively, with retention of configuration at C-2. [See also EC 3.8.1.2 (*S*)-2-haloacid dehalogenase, EC 3.8.1.9 (*R*)-2-haloacid dehalogenase and EC 3.8.1.10 2-haloacid dehalogenase (configuration-inverting)] |
| 3.8.1.2 | **(*S*)-2-Haloacid dehalogenase** | IUBMB comments: Acts on acids of short chain lengths, C2 to C4, with inversion of configuration at C-2. [See also EC 3.8.1.9 (*R*)-2-haloacid dehalogenase, EC 3.8.1.10 2-haloacid dehalogenase (configuration-inverting) and EC 3.8.1.11 2-haloacid dehalogenase (configuration-retaining)]. KEGG: Chloroalkane and chloroalkene degradation, Chlorocyclohexane and chlorobenzene degradation. MetaCyc: 2-Chloroacrylate degradation I. |
| 3.8.1.3 | **Haloacetate dehalogenase** | KEGG: Chloroalkane and chloroalkene degradation, Chlorocyclohexane and chlorobenzene degradation. MetaCyc: 1,2-Dichloroethane degradation, butachlor degradation, fluoroacetate degradation. |
| 3.8.1.4 | Thyroxine 5'-deiodinase | Transferred to [EC 1.97.1.10](https://www.brenda-enzymes.org/enzyme.php?ecno=1.97.1.10) |
| 3.8.1.5 | **Haloalkane dehalogenase** | IUBMB comments: Acts on a wide range of 1-haloalkanes, haloalcohols, haloalkenes and some haloaromatic compounds. KEGG: Chloroalkane and chloroalkene degradation, Chlorocyclohexane and chlorobenzene degradation. MetaCyc: 1,2-Dichloroethane degradation, gamma-hexachlorocyclohexane degradation. |
| 3.8.1.6 | [**4-Chlorobenzoate dehalogenase**](https://www.brenda-enzymes.org/enzyme.php?ecno=3.8.1.6#reactschemes) | IUBMB comments: Catalyses the first step in the degradation of chlorobenzoate in *Pseudomonas*. In many microorganisms, this activity comprises three separate enzymes, EC 6.2.1.33 (4-chlorobenzoate---CoA ligase), EC 3.8.1.7 (4-chlorobenzoyl-CoA dehalogenase) and EC 3.1.2.23 (4-hydroxybenzoyl-CoA thioesterase). |
| 3.8.1.7 | **4-Chlorobenzoyl-CoA dehalogenase** | IUBMB comments: Specific for dehalogenation at the 4-position. Can dehalogenate substrates bearing fluorine, chlorine, bromine and iodine in the 4-position. This enzyme is part of the bacterial 2,4-dichlorobenzoate degradation pathway. KEGG: Fluorobenzoate degradation. MetaCyc: 4-Chlorobenzoate degradation. |
| 3.8.1.8 | **Atrazine chlorohydrolase** | IUBMB comments: Involved in the degradation of the herbicide atrazine, 2-chloro-4-(ethylamino)-6-(isopropylamino)-1,3,5-triazine, in bacteria. KEGG: Atrazine degradation. MetaCyc: Atrazine degradation I (aerobic), atrazine degradation III. |
| 3.8.1.9 | **(*R*)-2-Haloacid dehalogenase** | IUBMB comments: Acts on acids of short chain lengths, C2 to C4, with inversion of configuration at C-2. [See also EC 3.8.1.2 (*S*)-2-haloacid dehalogenase, EC 3.8.1.10 2-haloacid dehalogenase (configuration-inverting) and EC 3.8.1.11 2-haloacid dehalogenase (configuration-retaining)]. |
| 4.1.1.77 | 2-Oxo-3-hexenedioate decarboxylase | MetaCyc: 3-Chlorocatechol degradation III (meta pathway), 4-chloronitrobenzene degradation. |
| 4.1.3.39 | [4-Hydroxy-2-oxovalerate aldolase](https://www.brenda-enzymes.org/enzyme.php?ecno=4.1.3.39#reactschemes) | KEGG: Dioxin degradation (4-chlorobiphenyl, PCB, PCDD, and PCDF degradation). |
| 4.1.3.40 | *Chorismate lyase* | MetaCyc: Polybrominated dihydroxylated diphenyl ethers biosynthesis, polybrominated phenols biosynthesis, spongiadioxin C biosynthesis. |
| 4.2.1.112 | Acetylene hydratase | KEGG: Chloroalkane and chloroalkene degradation. |
| 4.2.1.80 | 2-Oxopent-4-enoate hydratase | KEGG: Chlorinated Dioxin degradation. MetaCyc: 4-Chloronitrobenzene degradation. |
| 4.2.1.84 | Nitrile hydratase | KEGG: Fluorobenzoate and Bromoxynil degradation. |
| 4.2.3.135 | *DELTA6-protoilludene synthase* | IUBMB comments: Isolated from the fungus *Armillaria gallica*. Delta6-Protoilludene is the first step in the biosynthesis of the melleolides. MetaCyc: 6'-Dechloromelleolide F biosynthesis. |
| 4.3.3.5 | *4'-Demethylrebeccamycin synthase* | IUBMB comments: This enzyme catalyses a step in the biosynthesis of rebeccamycin, an indolocarbazole alkaloid produced by the bacterium *Lechevalieria aerocolonigenes*. The enzyme is a glycosylase, and acts in the reverse direction to that shown. It has a wide substrate range, and was shown to glycosylate several substrates, including the staurosporine aglycone, EJG-III-108A, J-104303, 6-N-methyl-arcyriaflavin C and indolo-[2,3-a]-carbazole [1,2]. MetaCyc: Rebeccamycin biosynthesis. |
| 4.4.1.23 | 2-Hydroxypropyl-CoM lyase | IKEGG: Chloroalkane and chloroalkene degradation. MetaCyc: Ethene and chloroethene degradation. |
| 4.5.1.1 | **DDT-Dehydrochlorinase** | KEGG: Dioxin degradation (dichlorodiphenyltrichloroethane degradation). |
| 4.5.1.2 | **3-Chloro-D-alanine dehydrochlorinase** | IUBMB comments: A pyridoxal-phosphate protein. The enzyme cleaves a carbon-chlorine bond, releasing a chloride and an unstable enamine product that tautomerizes to an imine form, which undergoes a hydrolytic deamination to form pyruvate and ammonia. The latter reaction, which can occur spontaneously, can also be catalysed by EC 3.5.99.10, 2-iminobutanoate/2-iminopropanoate deaminase. The enzyme's activity can also result in beta-replacement reactions, e.g. in the presence of hydrogen sulfide it can convert 3-chloro-D-alanine into D-cysteine and chloride. |
| 4.5.1.3 | **Dichloromethane dehalogenas**e | KEGG: Chloroalkane and chloroalkene degradation. |
| 4.5.1.4 | **L-2-Amino-4-chloropent-4-enoate dehydrochlorinase** | No comments. Reaction Scheme: L-2-amino-4-chloropent-4-enoate + [H_2_O](https://www.brenda-enzymes.org/ligand.php?brenda_ligand_id=1) = [2-oxopent-4-enoate](https://www.brenda-enzymes.org/ligand.php?brenda_ligand_id=5101)+[chloride](https://www.brenda-enzymes.org/ligand.php?brenda_ligand_id=298) + [NH_3_](https://www.brenda-enzymes.org/ligand.php?brenda_ligand_id=27) |
| 5.2.1.10 | 2-Chloro-4-carboxymethylenebut-2-en-1,4-olide isomerase | KEGG: Chlorocyclohexane and chlorobenzene degradation. MetaCyc: 3,5-Dichlorocatechol degradation. |
| 5.3.2.6 | 2-Hydroxymuconate tautomerase | KEGG: Dioxin degradation (PCDD degradation). MetaCyc: 3-Chlorocatechol degradation III (meta pathway), 4-chloronitrobenzene degradation. |
| 5.4.4.1 | (Hydroxyamino)benzene mutase | MetaCyc: 4-Chloronitrobenzene degradation. |
| 5.4.99.5 | *Chorismate mutase* | MetaCyc: Salinosporamide A biosynthesis. |
| 5.4.99.67 | [*4-Amino-4-deoxychorismate mutase*](https://www.brenda-enzymes.org/enzyme.php?ecno=5.4.99.67#reactschemes) | IUBMB comments: The enzyme, characterized from the bacteria *Streptomyces venezuelae* and *Streptomyces pristinaespiralis*, participates in the biosynthesis of the antibiotics chloramphenicol and pristinamycin IA, respectively. cf. EC 5.4.99.5, chorismate mutase. MetaCyc: [Chloramphenicol biosynthesis](about:blank). |
| 5.5.1.1 | Muconate cycloisomerase | IUBMB comments: Requires Mn^2+.^ Also acts (in the reverse reaction) on 3-methyl-cis,cis-muconate and, very slowly, on cis,trans-muconate. Not identical with EC 5.5.1.7 (chloromuconate cycloisomerase) or EC 5.5.1.11 (dichloromuconate cycloisomerase). KEGG: Chlorocyclohexane and chlorobenzene degradation, Fluorobenzoate degradation. |
| 5.5.1.11 | Dichloromuconate cycloisomerase | IUBMB comments: Requires Mn^2+^. The product of cycloisomerization of dichloro-cis,cis-muconate spontaneously eliminates chloride to produce cis-4-carboxymethylene-3-chlorobut-2-en-4-olide. Also acts, in the reverse direction, on cis,cis-muconate and its monochloro-derivatives, but with lower affinity. Not identical with EC 5.5.1.1 (muconate cycloisomerase) or EC 5.5.1.7 (chloromuconate cycloisomerase). KEGG: Chlorocyclohexane and chlorobenzene degradation. MetaCyc: 1,4-Dichlorobenzene degradation, 3,5-dichlorocatechol degradation. |
| 5.5.1.7 | Chloromuconate cycloisomerase | IUBMB comments: Requires Mn^2+^. The product of cycloisomerization of 3-chloro-cis,cis-muconate spontaneously eliminates chloride to produce cis-4-carboxymethylenebut-2-en-4-olide. Also acts on 2-chloro-cis,cis-muconate. Not identical with EC 5.5.1.1 (muconate cycloisomerase) or EC 5.5.1.11 (dichloromuconate cycloisomerase). KEGG: Chlorocyclohexane and chlorobenzene degradation, Fluorobenzoate degradation. MetaCyc: 2,4-Dichlorotoluene degradation, 2,5-dichlorotoluene degradation, 3,4-dichlorotoluene degradation, 3-chlorocatechol degradation I (ortho), 3-chlorocatechol degradation II (ortho), 4,5-dichlorocatechol degradation, 4-chlorocatechol degradation, 4-methylcatechol degradation (ortho cleavage), 5-chloro-3-methyl-catechol degradation, chlorosalicylate degradation. |
| 6.2.1.25 | Benzoate-CoA ligase | IUBMB comments: Also acts on 2-, 3- and 4-fluorobenzoate, but only very slowly on the corresponding chlorobenzoates. |
| 6.2.1.33 | 4-Chlorobenzoate-CoA ligase | IUBMB comments: Requires Mg^2+^. This enzyme is part of the bacterial 2,4-dichlorobenzoate degradation pathway. KEGG: Fluorobenzoate degradation. MetaCyc: 4-Chlorobenzoate degradation. |
| 6.2.1.53 | *L-Proline-[L-prolyl-carrier protein] ligase* | IUBMB comments: The enzyme participates in the biosynthesis of several pyrrole-containing compounds, such as undecylprodigiosin, prodigiosin, pyoluteorin, and coumermycin A1. It catalyses the activation of L-proline to an adenylate form, followed by its transfer to the 4'-phosphopantheine moiety of an L-prolyl-carrier protein. MetaCyc: Brominated pyrroles biosynthesis. |
| 6.3.4.6 | Urea carboxylase | KEGG: Atrazine degradation. |

*****BRENDA-EC42 functions are highlighted in bold (halogenases in italics and dehalogenases underlined).

**^†^**Most of the listed EC numbers were associated with oxidoreductase (92) followed by hydrolases (28), transferases (16), lyases (13), isomerases (8) and finally ligases (4).

**^§^**Based on IUBMB (International Union of Biochemistry and Molecular Biology), KEGG (Kyoto Encyclopedia of Genes and Genome), MetaCyc (Metabolic Pathways From all Domains of Life) and UniProtKB (UniProt Knowledgebase) recommended comments, most of the enzymes are involved in the degradation reactions of chloroalkane, chloroalkene, and different halogenated aromatic molecules containing the benzene ring. Albeit to a lesser extent, enzymes involved in biosynthetic pathways of halogenated antibiotics or polybrominated and chlorinated natural compounds as well as in pesticide (e.g. chlordecone, pyrethroid, chlorpyrifos) and herbicide (e.g. chloridazon, mecoprop, dichlorprop, alachlor, acetochlor, butachlor, atrazine, bromoxynil) degradation reactions were also retrieved.

**Table S2** BRENDA-EC161***** and -EC42**^‡^** functions annotated for each macroalgal metagenome

| Class of enzyme | BRENDA EC number | Function | Macroalgal  metagenome | *Sc* | *At* | *Hs* | *Total genes* |
| --- | --- | --- | --- | --- | --- | --- | --- |
| Oxidoreductases (EC 1.X.X.X)  -Total retrieved EC numbers: 41  -Total annotated genes: 2 080 | 1.1.1.1 | Alcohol dehydrogenase | *Sc-At-Hs* | 159 | 68 | 54 | 281 |
|  | 1.1.1.404 | Tetrachlorobenzoquinone reductase | *At-Hs* | 0 | 1 | 2 | 3 |
|  | 1.1.1.90 | Aryl-alcohol dehydrogenase | *Sc-At-Hs* | 26 | 2 | 10 | 38 |
|  | 1.1.2.7 | Methanol dehydrogenase (cytochrome c) | *Sc-At-Hs* | 3 | 3 | 2 | 8 |
|  | 1.1.2.8 | Alcohol dehydrogenase (cytochrome c) | *Sc-At-Hs* | 13 | 11 | 3 | 27 |
|  | 1.1.5.12 | D-Lactate dehydrogenase (quinone) | *Sc-At-Hs* | 7 | 6 | 6 | 19 |
|  | **1.11.1.10** | ***Chloride peroxidase*** | *Sc-At-Hs* | 6 | 5 | 1 | 12 |
|  | **1.11.1.18** | ***Bromide peroxidase*** | *Sc-At-Hs* | 11 | 9 | 5 | 25 |
|  | 1.13.11.2 | Catechol 2,3-dioxygenase | *Sc-At-Hs* | 22 | 10 | 9 | 41 |
|  | 1.13.11.37 | Hydroxyquinol 1,2-dioxygenase | *Sc-At-Hs* | 17 | 18 | 10 | 45 |
|  | 1.13.11.39 | Biphenyl-2,3-diol 1,2-dioxygenase | *Sc-At-Hs* | 24 | 19 | 9 | 52 |
|  | 1.14.11.43 | (*S*)-Dichlorprop dioxygenase (2-oxoglutarate) | *Sc-At-Hs* | 18 | 3 | 5 | 26 |
|  | 1.14.11.44 | (*R*)-Dichlorprop dioxygenase (2-oxoglutarate) | *Sc-At-Hs* | 85 | 26 | 17 | 128 |
|  | **1.14.12.13** | **2-Halobenzoate 1,2-Dioxygenase** | *Sc* | 2 | 0 | 0 | 2 |
|  | 1.14.12.3 | Benzene 1,2-dioxygenase | *Sc-At* | 1 | 1 | 0 | 2 |
|  | 1.14.13.1 | Salicylate 1-monooxygenase | *Sc* | 2 | 0 | 0 | 2 |
|  | 1.14.13.2 | 4-Hydroxybenzoate 3-monooxygenase† | Sc-At-Hs | 31 | 18 | 16 | 65 |
|  | 1.14.13.20 | 2,4-Dichlorophenol 6-monooxygenase | *Sc-At-Hs* | 8 | 7 | 5 | 20 |
|  | 1.14.13.24 | 3-Hydroxybenzoate 6-monooxygenase | *Sc-At-Hs* | 27 | 18 | 7 | 52 |
|  | **1.14.13.50** | **Pentachlorophenol 4-monooxygensase** | *Sc-At-Hs* | 3 | 3 | 2 | 8 |
|  | 1.14.15.23 | Chloroacetanilide N-alkylformylase | *Sc-At-Hs* | 1 | 1 | 2 | 4 |
|  | **1.14.19.49** | ***Tetracycline 7-halogenase*** | *Sc-Hs* | 16 | 0 | 2 | 18 |
|  | **1.14.19.9** | ***Tryptophan 7-halogenase*** | *Sc-At-Hs* | 35 | 115 | 62 | 212 |
|  | 1.18.1.3 | Ferredoxin-NAD+ reductase | *Sc-At-Hs* | 35 | 23 | 6 | 64 |
|  | 1.18.6.1 | Nitrogenase | *Sc-At-Hs* | 5 | 4 | 1 | 10 |
|  | 1.2.1.28 | Benzaldehyde dehydrogenase (NAD+) | *Sc-At-Hs* | 4 | 7 | 4 | 15 |
|  | 1.2.1.3 | Aldehyde dehydrogenase (NAD+) | *Sc-At-Hs* | 68 | 31 | 28 | 127 |
|  | 1.2.1.32 | Aminomuconate-semialdehyde dehydrogenase | *Sc-At-Hs* | 43 | 33 | 13 | 89 |
|  | 1.2.1.4 | Aldehyde dehydrogenase (NADP+) | *Sc-At-Hs* | 1 | 1 | 1 | 3 |
|  | 1.2.1.46 | Formaldehyde dehydrogenase | *Sc* | 7 | 0 | 0 | 7 |
|  | 1.2.1.5 | Aldehyde dehydrogenase [NAD(P)+] | *Sc-At-Hs* | 50 | 41 | 14 | 105 |
|  | 1.2.1.69 | *Fluoroacetaldehyde dehydrogenase* | *At* | 0 | 2 | 0 | 2 |
|  | **1.21.4.5** | **Tetrachlorohydroquinone reductive dehalogenase** | *At-Hs* | 0 | 1 | 1 | 2 |
|  | 1.21.98.2 | *Dichlorochromopyrrolate synthase* | *Sc* | 1 | 0 | 0 | 1 |
|  | 1.3.1.103 | 2-Haloacrylate reductase | *Sc-At-Hs* | 80 | 52 | 25 | 157 |
|  | 1.3.1.32 | Maleylacetate reductase | *Sc-At-Hs* | 7 | 4 | 4 | 15 |
|  | 1.3.8.14 | *L-Prolyl-[peptidyl-carrier protein] dehydrogenase* | *Sc-At-Hs* | 55 | 18 | 20 | 93 |
|  | 1.4.3.23 | *7-Chloro-L-tryptophan oxidase* | *Sc-At-Hs* | 7 | 8 | 2 | 17 |
|  | 1.6.5.7 | 2-Hydroxy-1,4-benzoquinone reductase | *Sc-At-Hs* | 9 | 4 | 6 | 19 |
|  | 1.7.1.16 | Nitrobenzene nitroreductase | *Sc-At-Hs* | 63 | 45 | 16 | 124 |
|  | 1.8.5.7 | Glutathionyl-hydroquinone reductase | *Sc-At-Hs* | 80 | 35 | 25 | 140 |
| Transferases (EC 2.X.X.X)  -Total retrieved EC numbers: 7  -Total annotated genes: 718 | 2.1.1.164 | *Demethylrebeccamycin-D-glucose O-Methyltransferase* | *Sc-At-Hs* | 3 | 1 | 3 | 7 |
|  | 2.2.1.8 | *Fluorothreonine transaldolase* | *Sc-At* | 7 | 3 | 0 | 10 |
|  | 2.3.1.28 | Chloramphenicol O-acetyltransferase | *Sc-At-Hs* | 22 | 17 | 22 | 61 |
|  | 2.4.2.1 | *Purine-nucleoside phosphorylase* | *Sc-At-Hs* | 128 | 86 | 89 | 303 |
|  | 2.5.1.18 | Glutathione transferase | *Sc-At-Hs* | 141 | 103 | 49 | 293 |
|  | **2.5.1.63** | ***Adenosyl-fluoride synthase*** | *Sc-At-Hs* | 6 | 5 | 7 | 18 |
|  | **2.5.1.94** | ***Adenosyl-chloride synthase*** | *Sc-At-Hs* | 14 | 9 | 3 | 26 |
| Hydrolases (EC 3.X.X.X)  -Total retrieved EC numbers: 18  -Total annotated genes: 1 323 | 3.1.1.45 | Carboxymethylenebutenolidase | *Sc-At-Hs* | 27 | 11 | 6 | 44 |
|  | 3.1.1.88 | Pyrethroid hydrolase | *Sc-At-Hs* | 28 | 3 | 4 | 35 |
|  | 3.1.2.23 | 4-hydroxybenzoyl-CoA thioesterase | *Sc-At* | 1 | 3 | 0 | 4 |
|  | 3.1.2.29 | Fluoroacetyl-CoA thioesterase | *Sc-At-Hs* | 4 | 1 | 2 | 7 |
|  | 3.3.2.10 | Soluble epoxide hydrolase | *Sc-At-Hs* | 67 | 35 | 24 | 126 |
|  | 3.5.1.131 | 1-Carboxybiuret hydrolase | *Sc-At-Hs* | 1 | 1 | 2 | 4 |
|  | 3.5.1.54 | Allophanate hydrolase | *Sc-At-Hs* | 11 | 10 | 10 | 31 |
|  | 3.5.2.15 | Cyanuric acid amidohydrolase | *Sc* | 2 | 0 | 0 | 2 |
|  | 3.5.4.42 | N-isopropylammelide isopropylaminohydrolase | *Sc-At* | 3 | 1 | 0 | 4 |
|  | 3.5.4.43 | Hydroxydechloroatrazine ethylaminohydrolase | *Sc-At-Hs* | 5 | 2 | 2 | 9 |
|  | 3.5.4.45 | Melamine deaminase | *Sc-At* | 1 | 1 | 0 | 2 |
|  | 3.5.99.11 | 2-Aminomuconate deaminase (2-hydroxymuconate-forming) | *Sc* | 3 | 0 | 0 | 3 |
|  | 3.5.99.5 | 2-Aminomuconate deaminase | *Sc-At-Hs* | 56 | 41 | 30 | 127 |
|  | **3.8.1.2** | **(*S*)-2-Haloacid dehalogenase** | *Sc-At-Hs* | 73 | 50 | 38 | 161 |
|  | **3.8.1.3** | **Haloacetate dehalogenase** | *Sc-At-Hs* | 45 | 25 | 13 | 83 |
|  | **3.8.1.5** | **Haloalkane dehalogenase** | *Sc-At-Hs* | 321 | 178 | 121 | 620 |
|  | **3.8.1.7** | **4-Chlorobenzoyl-CoA dehalogenase** | *Sc-At-Hs* | 20 | 6 | 9 | 35 |
|  | **3.8.1.8** | **Atrazine chlorohydrolase** | *Sc-At-Hs* | 12 | 7 | 7 | 26 |
| Lyases (EC 4.X.X.X)  -Total retrieved EC numbers: 8  -Total annotated genes: 392 | 4.1.1.77 | 2-Oxo-3-hexenedioate decarboxylase | *Sc-At-Hs* | 3 | 6 | 3 | 12 |
|  | 4.1.3.40 | *Chorismate lyase* | *Sc-At-Hs* | 13 | 12 | 20 | 45 |
|  | 4.2.1.112 | Acetylene hydratase | *Sc-At-Hs* | 15 | 9 | 3 | 27 |
|  | 4.2.1.80 | 2-Oxopent-4-enoate hydratase | *Sc-At-Hs* | 51 | 34 | 20 | 105 |
|  | 4.2.1.84 | Nitrile hydratase | *Sc-At-Hs* | 82 | 41 | 16 | 139 |
|  | 4.3.3.5 | *4'-Demethylrebeccamycin synthase* | *Sc-At-Hs* | 4 | 2 | 2 | 8 |
|  | 4.4.1.23 | 2-Hydroxypropyl-CoM lyase | *Sc-At-Hs* | 9 | 10 | 10 | 29 |
|  | **4.5.1.3** | **Dichloromethane dehalogenas**e | *Sc-At-Hs* | 12 | 12 | 3 | 27 |
| Isomerases (EC 5.X.X.X)  -Total retrieved EC numbers: 5  -Total annotated genes: 43 | 5.3.2.6 | 2-Hydroxymuconate tautomerase | *Sc* | 2 | 0 | 0 | 2 |
|  | 5.4.4.1 | (Hydroxyamino)benzene mutase | *Sc* | 1 | 0 | 1 | 2 |
|  | 5.4.99.5 | *Chorismate mutase* | *Sc-At-Hs* | 14 | 3 | 4 | 21 |
|  | 5.5.1.1 | Muconate cycloisomerase | *Sc-At-Hs* | 5 | 3 | 3 | 11 |
|  | 5.5.1.7 | Chloromuconate cycloisomerase | *Sc-At* | 2 | 5 | 0 | 7 |
| Ligases (EC 6.X.X.X)  -Total retrieved EC numbers: 2  -Total annotated genes: 91 | 6.2.1.25 | Benzoate-CoA ligase | *Sc-At-Hs* | 41 | 22 | 16 | 79 |
|  | 6.2.1.33 | 4-Chlorobenzoate-CoA ligase | *Sc-At-Hs* | 7 | 2 | 3 | 12 |
|  |  |  |  | **2 294** | **1 413** | **940** | **4 647** |

*Annotated genes involved in halogenation pathways are in italics (17 out of 81).

^‡^Halogenases and dehalogenases are highlighted in bold (BRENDA-EC42 functions). Dehalogenases are also underlined.

^†^This enzyme is also involved in a degradation pathway (Table S1).

*Sc*, *Sphaerococcus coronopifolius*; *At*, *Asparagopsis taxiformis*; *Hs*, *Halopteris scoparia*.
